# Global trends and inequities in smoking-attributable aortic aneurysm burden from 1990 to 2021 with future projections

**DOI:** 10.3389/fpubh.2025.1652544

**Published:** 2025-07-29

**Authors:** Shuai Zhang, Zhaohui Hua, Zhen Li, Hui Cao, Shuai Cheng

**Affiliations:** Department of Endovascular Surgery, The First Affiliated Hospital of Zhengzhou University, Zhengzhou, China

**Keywords:** aortic aneurysm, smoking-attributable burden, global trends, burden of disease, epidemiological forecasting

## Abstract

**Introduction:**

Aortic aneurysm (AA) remains a critical global health challenge, with smoking identified as a major modifiable risk factor contributing to its morbidity and mortality. Despite advancements in screening and treatment, the absolute burden of AA has risen significantly, particularly in aging populations and regions with socioeconomic disparities. This study leverages data from the Global Burden of Disease Study to analyze trends in smoking-related AA burden from 1990 to 2021, focusing on mortality, disability-adjusted life years (DALYs), and socioeconomic determinants.

**Methods:**

Using GBD 2021 data, we assessed age-standardized mortality rates (ASMR), age-standardized DALY rates (ASDR). Joinpoint regression identified trend inflection points, validated via grid search and Monte Carlo permutation tests, with annual percent change (APC) quantified. Age-period-cohort modeling was analyzed effects in populations aged ≥30 years (5-year age intervals). Decomposition analysis partitioned contributions of population growth, aging, and epidemiological factors. Spearman’s correlation linked the Sociodemographic Index (SDI) to AA burden. ARIMA modeling projected trends to 2022–2036.

**Results:**

Despite a significant global decline in age-standardized mortality and DALYs, the absolute burden of smoking-related AA has increased, with marked disparities by sex, age, and socioeconomic development. Males consistently exhibited higher mortality and DALYs than females, and the older adults remained the most affected. Joinpoint regression and age-period-cohort modeling revealed declining trends in high-income regions but rising burdens in low-SDI areas. Decomposition analysis identified population growth and aging as key drivers of increased mortality and DALYs, while epidemiological improvements partially offset these trends. Socioeconomic analysis showed a threshold effect: AA burden increased with SDI up to a point, then declined with further development, suggesting effective health systems and tobacco control policies play a crucial role. Forecasts using ARIMA modeling predict a continued global decline in ASMR and ASDR by 2036, though disparities will persist, especially in low-resource settings.

**Conclusion:**

These findings underscore the need for targeted, equity-focused tobacco control and vascular health interventions to mitigate the evolving global impact of smoking-related AA.

## 1 Introduction

Aortic aneurysms (AA) represent a significant public health concern due to their silent progression and high mortality upon rupture (1). As localized dilations of the aorta, these aneurysms, particularly thoracic and abdominal aortic aneurysms (AAAs), are often asymptomatic until catastrophic events occur, contributing to substantial years of life lost and disability-adjusted life years (DALYs) globally (2). AAA represents a prototypical age-related vascular disorder, with prevalence escalating from 1.2–4% in adults aged ≥50 years to 8% in men >65 years, predominantly those with hypertension or cardiovascular comorbidities, particularly among aging populations and those with identifiable modifiable risk factors (3–5). Despite advancements in imaging and surgical interventions, the overall global burden remains substantial, with significant geographical disparities and demographic variations.

Among the modifiable risk factors, smoking has been identified as the most consistent contributor to the development and progression of aortic aneurysms (6, 7). The pro-inflammatory and proteolytic effects of tobacco smoke on vascular walls accelerate medial degeneration and aortic dilatation, significantly elevating the risk of aneurysm formation and rupture (8, 9). As smoking remains prevalent in many parts of the world, its contribution to the disease burden of aortic aneurysms is both critical and preventable. Furthermore, the interaction between smoking and other comorbidities, such as hypertension and hyperlipidemia, further compounds the vascular risk profile (10). Smoking contributes substantially to the cardiovascular disease (CVD) burden, causing an estimated 2.7 million deaths and 71.4 million DALYs in 2019 (11). A meta-analysis of 18 cohort studies involving 15,475 individuals diagnosed with small aortic aneurysms revealed substantial heterogeneity in smoking prevalence, with current smoking rates ranging from 18 to 60% across cohorts (12). While recent studies characterize all-cause or BMI-attributable AA burden (13–15), this is the first analysis to quantify the global smoking-attributable AA burden using GBD 2021 data, revealing unique socioeconomic and temporal patterns critical for tobacco control. Furthermore, the risk of AAA was fivefold higher in current smokers and twofold higher in former smokers compared to never-smokers, underscoring smoking’s dose-dependent contribution to AA pathogenesis (16). There is a well-established pathophysiological basis for the relationship between smoking and aortic aneurysm development. Smoking contributes to endothelial dysfunction and promotes chronic vascular inflammation, which accelerates the degradation of extracellular matrix components such as elastin and collagen in the aortic wall (17). This is mediated primarily through increased expression of matrix metalloproteinases (MMPs), especially MMP-2 and MMP-9. Moreover, smoking induces oxidative stress and impairs the function of vascular smooth muscle cells, further weakening the structural integrity of the aortic wall (14). These biological effects collectively predispose individuals to aneurysm formation, enlargement, and rupture.

In light of these risks, smoking cessation has been prioritized in global tobacco control efforts, particularly under the WHO Framework Convention on Tobacco Control, which is a treaty mandating evidence-based population-level interventions to comprehensively mitigate both active and passive smoking exposure. Moreover, smoking cessation and avoidance of second-hand smoking is identified as a healthy lifestyle modification in patients with AAs by American College of Cardiology/American Heart Association (18). While Wang et al. (13) pioneered early-onset AA epidemiology, they did not quantify risk factor-attributable burdens. Jing et al. (14) established BMI as an AA risk driver but acknowledged smoking as the dominant yet unanalyzed factor. Tian et al. (15) projected all-cause AA burden but could not isolate preventable smoking-attributable deaths. Our study bridges these gaps by: (1) Focusing exclusively on smoking, responsible for >60% of AA DALYs vs. BMI’s 13% (GBD 2021); (2) Identifying an SDI 0.75 threshold where tobacco control efficacy reverses AA trends; (3) Providing sex-stratified, smoking-attributable projections to 2036 for precision policy. Despite the well-documented association between smoking and aortic aneurysms, there is limited comparative analysis of the global, regional, and national burden of this condition stratified by smoking status. The aim of this study is to comprehensively analyze the long-term trends and future burden of aortic aneurysm mortality attributable to smoking. Specifically, our objectives are: (1) to quantify temporal trends using joinpoint regression analysis; (2) to evaluate age, period, and cohort effects through APC modeling; (3) to identify contributing factors to mortality changes via decomposition analysis; and (4) to forecast future trends using ARIMA models. These methods collectively provide a multidimensional understanding of the impact of smoking on AA mortality. Our objective is to inform targeted prevention strategies and contribute to evidence-based policy-making aimed at reducing premature mortality from aortic aneurysms worldwide.

## 2 Materials and methods

### 2.1 Data sources and definitions

The data in this study were primarily derived from the Global Burden of Disease database, managed by the Institute for Health Metrics and Evaluation (IHME) at the University of Washington. The GBD provides comprehensive epidemiological data, spanning morbidity, mortality, and risk factor distributions globally, from 1990 to 2021. For this analysis, we focused on the data related to AA attributable to smoking exposure. The GBD study calculates smoking-attributable AA burden using comparative risk assessment (CRA) methodology. This involves estimating population attributable fractions (PAFs) based on: (1) pooled relative risks (RRs) of AA for smoking exposure derived from meta-analyses, and (2) exposure prevalence from population-based surveys. We directly extracted pre-calculated AA deaths and DALYs attributable to smoking from GBD outputs, no *de novo* PAF calculations were performed in this study (19). The GBD database includes extensive datasets from 204 countries and regions, which are categorized into five groups based on the Sociodemographic Index (SDI). The SDI, ranging from 0 to 1, reflects a country’s level of socio-economic development, with higher values indicating more advanced societal progress and, generally, better health outcomes. For this study, the GHDx analytical platform^1^ was employed to extract longitudinal data spanning the period 1990–2021. We specifically obtained records related to crude incidence rates, mortality figures, and age-standardized metrics, including age-standardized disability-adjusted life years (ASDR) and age-standardized mortality rates (ASMR). The age-standardization process controls for variations in population age structures across different regions and time periods, allowing for more accurate comparisons. For trend Analysis, the percentage change in disease burden attributable to smoking was calculated using the formula:

$$\text{Change Rate}=\frac{\text{Value in}\;2021-\text{Value in}\;1990}{\text{Value in}\;1990}\times 100\%$$

### 2.2 Joinpoint regression analysis

To analyze temporal trends in AA burden attributable to smoking, we applied joinpoint regression analysis, a technique that identifies changes in the trends of health outcomes over time. This method is especially valuable when analyzing complex datasets with non-linear trends, as it allows for the identification of points where significant changes (referred to as joinpoints) occur in the trend trajectory. Joinpoint regression fits multiple linear segments to the logarithmically transformed rates, capturing both broad trends and any abrupt shifts in the data. The optimal number and locations of joinpoints were determined using the grid search method (GSM) (20), which systematically explores different configurations of joinpoints. The model was further refined and validated using a Monte Carlo permutation test, ensuring statistical robustness in the detected trend shifts. Additionally, Annual Percent Change (APC) was calculated for each segment, providing a quantitative measure of the direction and magnitude of changes in the trends (21). Positive APC values indicate upward trends, while negative values suggest declines in AA burden. The Average Annual Percentage Change (AAPC) and APC were reported along with their 95% uncertainty intervals (UIs). Projections were visualized using curve plots. Extended Average Annual Percentage Change (EAPC): Long-term trends in ASMR and ASDR were quantified using the EAPC, calculated as follows:

Ln[R]=β0+β1·T

Where: *R* is the age-standardized rate in a specific year, *T* is the year (e.g., *T* = 0 for 1990), and *β*1 is the regression coefficient. The EAPC was derived as:

EAPC=(eβ1−1)×100%f(x)

If the 95% UI for EAPC was entirely positive, the trend was considered significantly increasing; if entirely negative, the trend was significantly decreasing; if the 95% UI included zero, the trend was considered non-significant.

### 2.3 Age-period-cohort model

Age-period-cohort models are commonly used to explore the impact of age, time period, and birth cohort on health outcomes, and have proven effective in the analysis of trends in DALYs and ortality. The model is grounded in Poisson distributions, but the linear relationship between age, period, and cohort poses significant challenges in estimating the independent effects of each factor. To overcome these challenges, various methodological innovations, including intrinsic stimators (22), penalty function methods (23), and other advanced techniques (24), have been developed.

In this study, we applied an Age-Period-Cohort model to examine the changes in AA burden, ocusing on DALYs and mortality across different age groups, time periods, and cohorts. However, due to data limitations, age-specific data were available only for individuals aged 30 years and older. Therefore, age groups were defined in 5-year intervals (e.g., 30–34, 35–39, …, 90–94, and 95+). Using the GBD dataset, we collected the total number of DALY cases and deaths within these age categories for the periods 1990–1994, 1995–1999, …, and 2017–2021. The Age-Period-Cohort model was fitted using the Epi package in R (version 4.4.2), which is specifically designed for epidemiological data analysis. Age-period-cohort modeling were employed to isolate smoking-specific drivers, overcoming limitations of aggregated risk-factor analyses in prior studies. This approach was employed to examine how AA burden attributable to smoking evolved within different age groups over time.

### 2.4 Decomposition analysis

Decomposition analysis was conducted to investigate the contributions of population size, population aging, and epidemiological factors to regional disparities in AA-related mortality and DALYs. The decomposition method, developed by Bashir and Das Gupta (25), was used to quantify how changes in these factors contribute to variations in the disease burden across regions. Cheng’s decomposition technique was used to disentangle the effects of demographic shifts (such as population aging) and epidemiological trends from the influence of population size on the observed disparities (26). This method provides valuable insights into how regional differences in AA burden attributable to smoking might be influenced by broader demographic and epidemiological factors. By analyzing the combined effects of population size, aging, and epidemiological changes, we are able to pinpoint the specific contributors to the rising or falling burden of AA in various regions. This detailed understanding is essential for developing targeted public health strategies aimed at mitigating the impact of smoking-related AA.

### 2.5 Correlation analysis

To explore the relationship between socioeconomic development and the burden of AA, correlation analysis was conducted between the SDI and both ASMR and ASDR associated with AA attributable to smoking. Spearman’s rank correlation coefficients (*ρ*) were computed to assess the strength and direction of these associations. This analysis provides insights into how changes in SDI, which reflects the level of societal development, influence the burden of AA across different countries and regions. Nonlinear associations between SDI and burden metrics were visualized using locally estimated scatterplot smoothing (LOESS). Threshold effects in the SDI-burden relationship were identified as inflection points where the direction of association reversed (e.g., from positive to negative). The approximate SDI value (0.75) representing this threshold was empirically determined through visual inspection of LOESS curves and confirmed by comparing model fit statistics across candidate inflection points. A positive correlation would suggest that higher SDI values are associated with a greater burden of AA, potentially due to higher smoking rates or better diagnostic and reporting systems. Conversely, a negative correlation might indicate that more developed regions have managed to reduce the burden through effective public health policies and interventions.

### 2.6 ARIMA model for forecasting

To predict future trends in the burden of AA attributable to smoking, we employed the Autoregressive Integrated Moving Average (ARIMA) model (27), a widely used method for time-series forecasting. The ARIMA model is characterized by three parameters: *p*, the autoregressive component; *d*, the degree of differencing; and *q*, the moving average component. We identified the above appropriate parameters (*p*, *d*, *q*), which were further refined by minimizing the Akaike Information Criterion (AIC). Residual diagnostics were performed to ensure white noise residuals and model adequacy. These parameters allow the model to capture the temporal dependencies in the data, providing a robust framework for forecasting future trends based on historical patterns. Using mortality and DALY data from 1990 to 2021, we fit an ARIMA model to project the burden of AA due to smoking for the period 2022–2036. The ARIMA projections assume continuity in historical trends of smoking prevalence, healthcare access, and AA management practices. They do not account for unforeseen disruptions (e.g., new tobacco products, pandemics, or policy shifts). Uncertainty prediction intervals (95% PIs) were generated via Monte Carlo simulations of residual errors to quantify stochastic variability around point estimates. This projection is essential for informing public health policy and resource allocation, helping stakeholders plan for the future impact of smoking-related AA.

## 3 Results

### 3.1 Global burden of smoking-attributable aortic aneurysm: rising mortality and DALYs amid declining age-standardized rates and persistent gender-age disparities

The global mortality associated with AA attributable to smoking has exhibited a notable upward trend, increasing from 36,485.01 (95% UI: 31,090.28–42,553.91) in 1990 to 47,537.63 (95% UI: 39,480.31–56,007.55) in 2021, reflecting an approximate 1.30-fold rise over the 31-year period. Similarly, the global burden of disease, as measured by DALYs due to AA linked to smoking, has also increased significantly, rising from 869,302.27 (95% UI: 754,922.33–1,000,697.19) in 1990 to 1,157,487.88 (95% UI: 989,965.60–1,325,831.15) in 2021, indicating a 1.33-fold escalation. Despite this overall increase in the burden of AA, ASMR per 100,000 people showed a downward trajectory, with an EAPC of −2.17 (95% UI: −2.30 to −2.05), suggesting a general reduction in mortality rates. Likewise, ASDR demonstrated a significant decline, with an EAPC of −1.90 (95% UI: −2.01 to −1.78) (Table 1).

**Table 1:** DALYs and mortality for AA attributable to smoking age-standardized rates with 95% uncertainty intervals and EAPC (estimated annual percent change) with 95% uncertainty intervals, 1990–2021.

Location	Deaths					DALYs				
	Number (95% UI)		ASMR (95% UI)		EAPC (95% UI)	Number (95% UI)		ASDR (95% UI)		EAPC (95% UI)
	1990	2021	1990	2021		1990	2021	1990	2021	
Global	36485.01 (31090.28–42553.91)	47537.63 (39480.31–56007.55)	0.98 (0.82–1.15)	0.56 (0.46–0.66)	−2.17 (−2.3 to −2.05)	869302.27 (754922.33–1000697.19)	1157487.88 (989965.6–1325831.15)	21.66 (18.72–24.96)	13.35 (11.4–15.31)	−1.9 (−2.01 to −1.78)
Sex										
Female	7111.44 (5824.25–8535.8)	8424.71 (6528.55–10745.98)	0.35 (0.29–0.42)	0.18 (0.14–0.23)	−2.49 (−2.64 to −2.33)	157197.53 (132749.41–184824.18)	185275.65 (149548.08–224721.02)	7.39 (6.21–8.73)	4.06 (3.29–4.91)	−2.29 (−2.42 to −2.15)
Male	29373.57 (25184.39–34223.93)	39112.92 (33109.64–45293.74)	1.82 (1.53–2.15)	1.01 (0.84–1.18)	−2.26 (−2.38 to −2.14)	712104.74 (615605.61–818975.38)	972212.23 (835139.76–1104759.28)	38.84 (33.4–45.13)	23.66 (20.24–26.93)	−1.93 (−2.04 to −1.82)
21 GBD regions										
Andean Latin America	49.17 (38.38–64.37)	117.29 (89.07–156.32)	0.24 (0.19–0.32)	0.2 (0.15–0.27)	−0.61 (−0.72 to −0.49)	1339.99 (1065.22–1723.56)	3069.35 (2410.73–4020.84)	6.05 (4.78–7.85)	5.01 (3.93–6.58)	−0.57 (−0.69 to −0.44)
Australasia	657.56 (545.88–776.93)	347.16 (269.94–445.85)	2.71 (2.25–3.2)	0.63 (0.5–0.8)	−5.14 (−5.31 to −4.98)	14314.24 (12222.33–16562.99)	7088.06 (5799.44–8712.53)	59.64 (51.01–68.87)	14.3 (11.88–17.22)	−5.04 (−5.21 to −4.87)
Caribbean	324.54 (265.72–385.4)	402.82 (318.22–502.26)	1.28 (1.05–1.54)	0.75 (0.59–0.93)	−2.19 (−2.41 to −1.97)	7263.95 (6121.44–8439.63)	8997.55 (7341.51–10931.19)	27.72 (23.25–32.29)	16.74 (13.66–20.33)	−2.05 (−2.29 to −1.82)
Central Asia	161.19 (136.52–196.87)	542.49 (447.91–637.99)	0.33 (0.28–0.4)	0.67 (0.56–0.79)	2.44 (2.22 to 2.66)	5049.48 (4308.14–6154.03)	15161.61 (12530.65–17838.56)	9.93 (8.47–12.1)	17.11 (14.18–20.14)	1.73 (1.53 to 1.94)
Central Europe	2054.39 (1807.03–2310.73)	2407.48 (2005.61–2828.05)	1.38 (1.21–1.55)	1.11 (0.93–1.3)	−1.01 (−1.23 to −0.8)	54801.69 (48776.19–60444.95)	58474.84 (49390.85–67863.63)	36.37 (32.44–40.11)	29.24 (24.89–33.71)	−1.02 (−1.23 to −0.8)
Central Latin America	391.02 (334.55–449.43)	732.52 (572.85–905.75)	0.48 (0.4–0.55)	0.29 (0.23–0.36)	−2.43 (−2.74 to −2.12)	10836.37 (9408.73–12220.71)	19084.98 (15250.59–23377.5)	11.99 (10.37–13.66)	7.41 (5.91–9.07)	−2.42 (−2.72 to −2.11)
Central Sub-Saharan Africa	89.91 (35.1–158.02)	192.35 (90.59–329.27)	0.4 (0.16–0.71)	0.34 (0.16–0.58)	−0.68 (−1.1 to −0.25)	2668.68 (1045.02–4661.74)	6023.57 (2828.25–10439.94)	10.47 (4.09–18.28)	9.05 (4.26–15.5)	−0.57 (−0.97 to −0.17)
East Asia	1305.66 (956.35–1776.35)	4623.42 (3421.34–6002.72)	0.15 (0.11–0.2)	0.22 (0.16–0.28)	1.58 (1.4 to 1.76)	43507.38 (31708.27–59986.62)	137466.63 (101252.76–179382.04)	4.31 (3.16–5.9)	6.60 (4.84–8.58)	1.60 (1.41 to 1.79)
Eastern Europe	2701.79 (2423.19–2981.76)	5126.26 (4364.07–5883.51)	0.96 (0.86–1.06)	1.5 (1.28–1.72)	1.15 (0.88 to 1.42)	79512.02 (71995.21–86901.42)	141839.36 (121389.4–161202.6)	28.49 (25.81–31.17)	43.68 (37.66–49.23)	1.03 (0.73 to 1.33)
Eastern Sub-Saharan Africa	256.87 (108.59–453.58)	550.89 (242.71–940.86)	0.37 (0.16–0.65)	0.33 (0.15–0.56)	−0.75 (−0.99 to −0.51)	7301.6 (3064.92–13204.42)	16657.31 (7242.27–28294.01)	8.95 (3.79–15.92)	8.33 (3.65–14.22)	−0.57 (−0.79 to −0.35)
High-income Asia Pacific	2166.65 (1858.21–2483.52)	5858.21 (4615.48–7252.07)	1.10 (0.94–1.27)	1.24 (1.02–1.47)	0.35 (0.21 to 0.48)	49328.79 (43273.52–55568.65)	113147.63 (94524.09–133713.52)	24.19 (21.17–27.32)	29.38 (25.23–33.62)	0.67 (0.55 to 0.79)
High-income North America	8250.67 (6951.61–9685.04)	4743.5 (3818.34–5782.67)	2.27 (1.93–2.66)	0.76 (0.62–0.91)	−4.25 (−4.52 to −3.97)	180501.04 (156348.7–206337.38)	112813.7 (95730.78–131766.09)	51.88 (45.19–59.01)	20.06 (17.3–23.03)	−3.71 (−3.99 to −3.44)
North Africa and Middle East	522.94 (361.14–793.23)	1498.9 (1196.61–1830.33)	0.3 (0.21–0.46)	0.33 (0.26–0.4)	0.29 (0.23 to 0.35)	16409.67 (11250.75–25104.42)	43784.5 (35227.19–53485.3)	8.38 (5.76–12.81)	8.44 (6.75–10.28)	0.02 (−0.05 to 0.08)
Oceania	17.68 (12.47–25.26)	38.53 (27.5–52.89)	0.59 (0.41–0.83)	0.48 (0.35–0.66)	−0.85 (−0.94 to −0.75)	573.42 (406.94–819.84)	1269.33 (896.79–1776.99)	16.11 (11.41–22.97)	13.57 (9.68–18.62)	−0.76 (−0.85 to −0.67)
South Asia	1147.33 (669.7–2367.61)	4298.52 (2808.91–7206.7)	0.22 (0.13–0.44)	0.31 (0.2–0.52)	1.17 (1.12 to 1.21)	31759.68 (18663.42–65852.15)	107748.39 (69115.9–181259.44)	5.17 (3.02–10.68)	7.07 (4.58–11.91)	0.97 (0.93 to 1)
Southeast Asia	759.1 (590.63–1049.79)	2374.32 (1902.92–2942.29)	0.35 (0.26–0.49)	0.41 (0.32–0.51)	0.27 (0.17 to 0.36)	19001.07 (15004.85–25989.34)	57346.71 (46311.89–70455.08)	7.39 (5.74–10.17)	8.7 (7.04–10.78)	0.34 (0.25 to 0.43)
Southern Latin America	791.79 (670.99–920.24)	716.07 (591.82–853.76)	1.69 (1.43–1.96)	0.83 (0.69–0.99)	−2.47 (−2.71 to −2.23)	21053.14 (18190.53–23938.67)	18678.31 (15891.88–21570.41)	44.6 (38.52–50.77)	22.37 (19.16–25.7)	−2.42 (−2.65 to −2.19)
Southern Sub-Saharan Africa	268.33 (208.07–334.84)	317.87 (256.98–381.78)	1.04 (0.78–1.34)	0.55 (0.44–0.66)	−2.73 (−3.03 to −2.43)	7442.88 (6064.06–8879.66)	9469.16 (7719.94–11145.5)	25.11 (19.98–30.53)	14.49 (11.74–17.3)	−2.36 (−2.64 to −2.08)
Tropical Latin America	1484.78 (1312.48–1656.74)	3605.76 (2995.31–4328.88)	1.60 (1.4–1.81)	1.40 (1.16–1.68)	−0.86 (−1.07 to −0.66)	43619.85 (39127.49–47859.95)	94970.87 (80972.15–110863.52)	43.06 (38.34–47.56)	36.07 (30.7–42.22)	−1.06 (−1.28 to −0.84)
Western Europe	12859.79 (10919.95–14883.87)	8544.97 (6904.86–10424.9)	2.15 (1.83–2.48)	0.89 (0.74–1.07)	−3.39 (−3.64 to −3.14)	266710.49 (233007.04–300530.63)	169368.37 (142587.2–199835.5)	46.92 (41.33–52.58)	20.36 (17.37–23.47)	−3.22 (−3.45 to −2.99)
Western Sub-Saharan Africa	223.83 (75.02–431.73)	498.3 (193.02–899.76)	0.27 (0.09–0.51)	0.25 (0.10–0.45)	−0.48 (−0.63 to −0.33)	6306.83 (2072.75–12139.57)	15027.65 (5728.72–26825.4)	6.6 (2.2–12.61)	6.46 (2.5–11.57)	−0.39 (−0.56 to −0.23)

**Figure 1:**
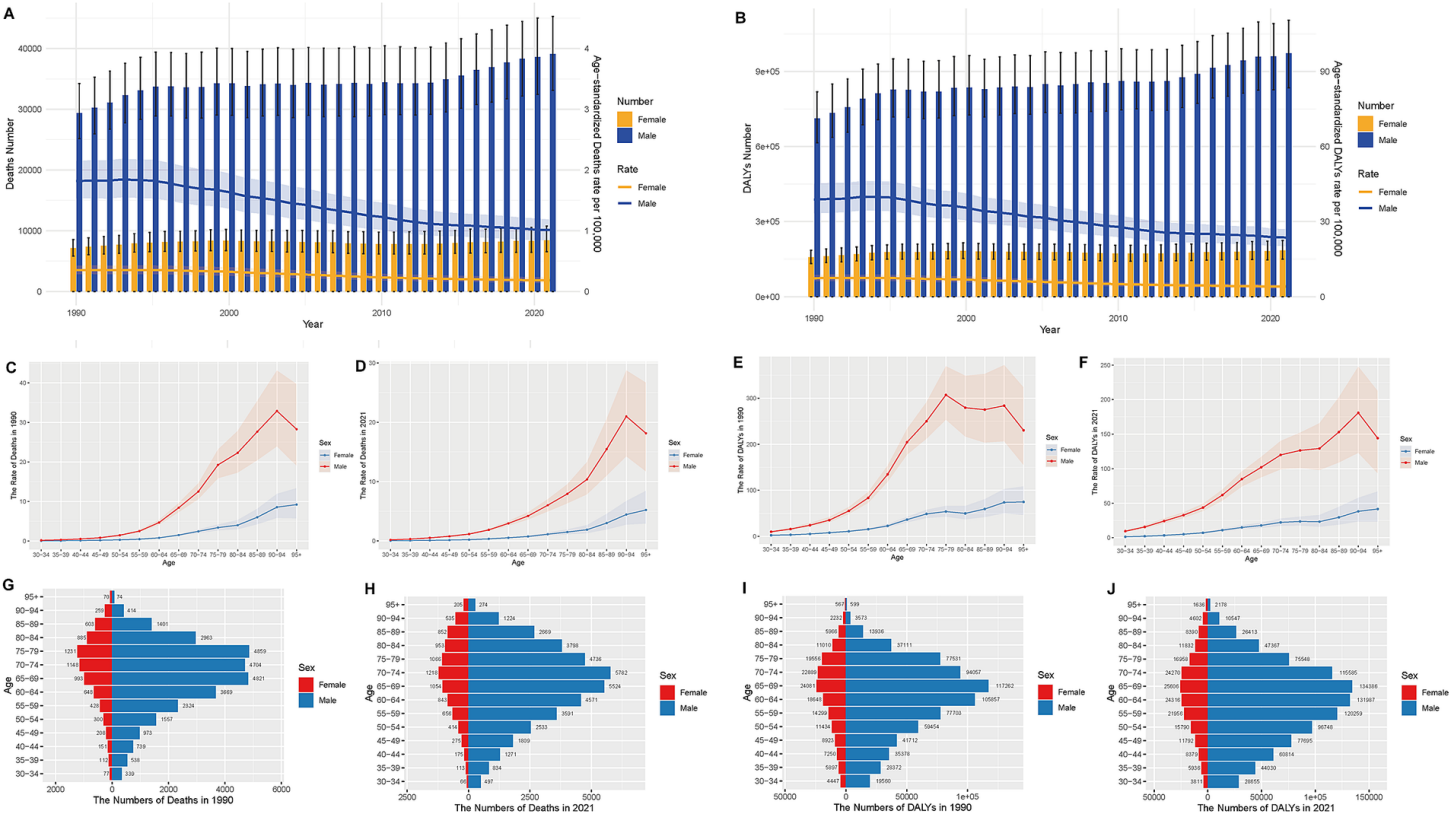
Trends in mortality rates and DALYs of aortic aneurysms attributable to smoking in 1990 and 2021. **(A)** Age distribution of DALYs by sex in 2021. Time trends of the burden of aortic aneurysms attributable to smoking from 1990 to 2021, including changes in absolute number of deaths and age-standardized mortality rates (ASMR); **(B)** as well as changes in DALYs and age-standardized DALY rates (ASDR); **(C)** Age-specific mortality rates in 1990; **(D)** Age-specific mortality rates in 2021; **(E)** Age-specific DALYs in 1990; **(F)** Age-specific DALYs in 2021; **(G)** Age distribution of deaths by sex in 1990; **(H)** Age distribution of deaths by sex in 2021; **(I)** Age distribution of DALYs by sex in 1990; **(J)** Age distribution of DALYs by sex in 2021.

Gender disparities in the mortality and DALYs burden of AA attributable to smoking persisted throughout the study period. In both 1990 and 2021, males consistently exhibited higher mortality and DALYs compared to females across all age groups, as shown in Figures 1A,B. Notably, while the burden in males decreased annually from 1990 to 2021, with both mortality and DALYs following a declining trend, females showed a relatively stable pattern in both mortality and DALYs during the same period. In 1990, the highest mortality was observed in the 90–94 age group among males, and this age group remained the highest in terms of mortality in 2021 (Figures 1C,D). Similarly, in 1990, the peak DALYs were seen in the 75–79 age group for males, whereas by 2021, the 90–94 age group emerged as the group with the highest DALYs (Figures 1E,F). For both mortality and DALYs, a general trend was observed wherein the numbers initially increased with age and reached a peak before gradually declining. In 1990, the highest mortality was observed in the 75–79 age group, followed by the 70–74 age group (Figures 1G,H). By 2021, the peak DALYs had shifted to the 65–69 age group, which remained the highest age group for DALYs in 2021 (Figures 1I,J). This shift indicates an evolving pattern in the age distribution of the disease burden, with younger populations beginning to exhibit more significant contributions to the overall burden of AA due to smoking.

### 3.2 Joinpoint regression reveals sex-specific and SDI-divergent trends in smoking-attributable aortic aneurysm burden: accelerated declines in high-income regions contrast with rising low-income burden

**Figure 2:**
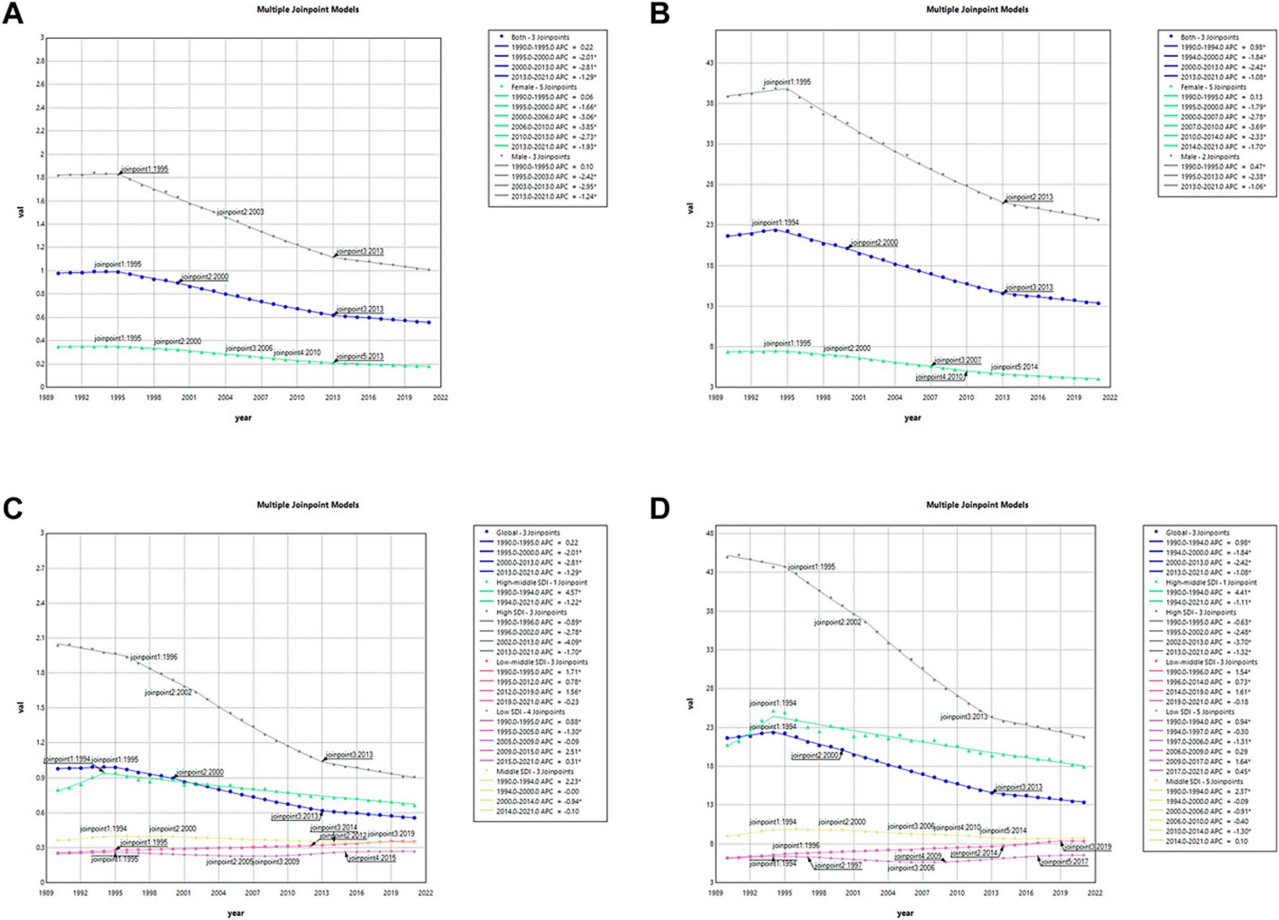
Trends in ASMR and ASDR in smoking-attributable aortic aneurysm burden. **(A)** ASMR attributable to smoking by gender in 1990; **(B)** ASDR attributable to smoking by gender in 1990; **(C)** ASMR attributable to smoking by different SDI in 1990; **(D)** ASMR attributable to smoking by different SDI in 2021.

A joinpoint regression analysis indicated a declining trend in both ASMR and ASDR of AA attributable to smoking across both sexes and the overall population (AAPC = −1.80, 95%UI: −1.89 to −1.72 for ASMR and AAPC = −1.53, 95%UI: −1.63 to −1.43 for ASDR, Supplementary Table S1). Notably, the most rapid decline in ASMR among males occurred between 1995 and 2013 (APC = −2.38, 95% UI: −2.44 to −2.32), while the steepest decrease among females was observed from 2007 to 2010 (APC = −3.69, 95% UI: −4.77 to −2.59, Figure 2A and Supplementary Table S1). Similarly, the ASDR of AA attributable to smoking exhibited the fastest reduction in males between 2003 and 2013 (APC = −2.95, 95% UI: −3.07 to −2.82) and in females from 2006 to 2010 (APC = −3.85, 95% UI: −4.34 to −3.36, Figure 2B and Supplementary Table S1). ASMR demonstrated the most notable decline occurring in High-middle SDI and High SDI (AAPC = -0.42, 95%UI: −0.63 to −0.21 and AAPC = −2.32, 95%UI: −2.43 to −2.21, respectively), however, significantly increase occurring in Low-middle SDI and Low SDI (AAPC = 0.97, 95%UI: 0.86–1.07 and AAPC = 0.21, 95%UI: 0.05–0.37, respectively). And for Low SDI, the most notable increase occurring between 2009 and 2015 (APC = 2.51, 95%UI: 2.18–2.84, Figure 2C and Supplementary Table S2). The ASDR also showed the steepest reduction observed during the same location (AAPC = −0.49, 95%UI: −0.67 to −0.31 for High-middle SDI; AAPC = −2.61, 95%UI: −2.71 to −2.51 for High SDI; and AAPC = −0.16, 95%UI: −0.28 to −0.05 for Middle SDI), and significantly increase occurring in Low-middle SDI and Low SDI (Figure 2D and Supplementary Table S2).

### 3.3 The burden of AA is attributable to smoking exposure by 21 GBD regions, and 204 countries and territories

**Figure 3:**
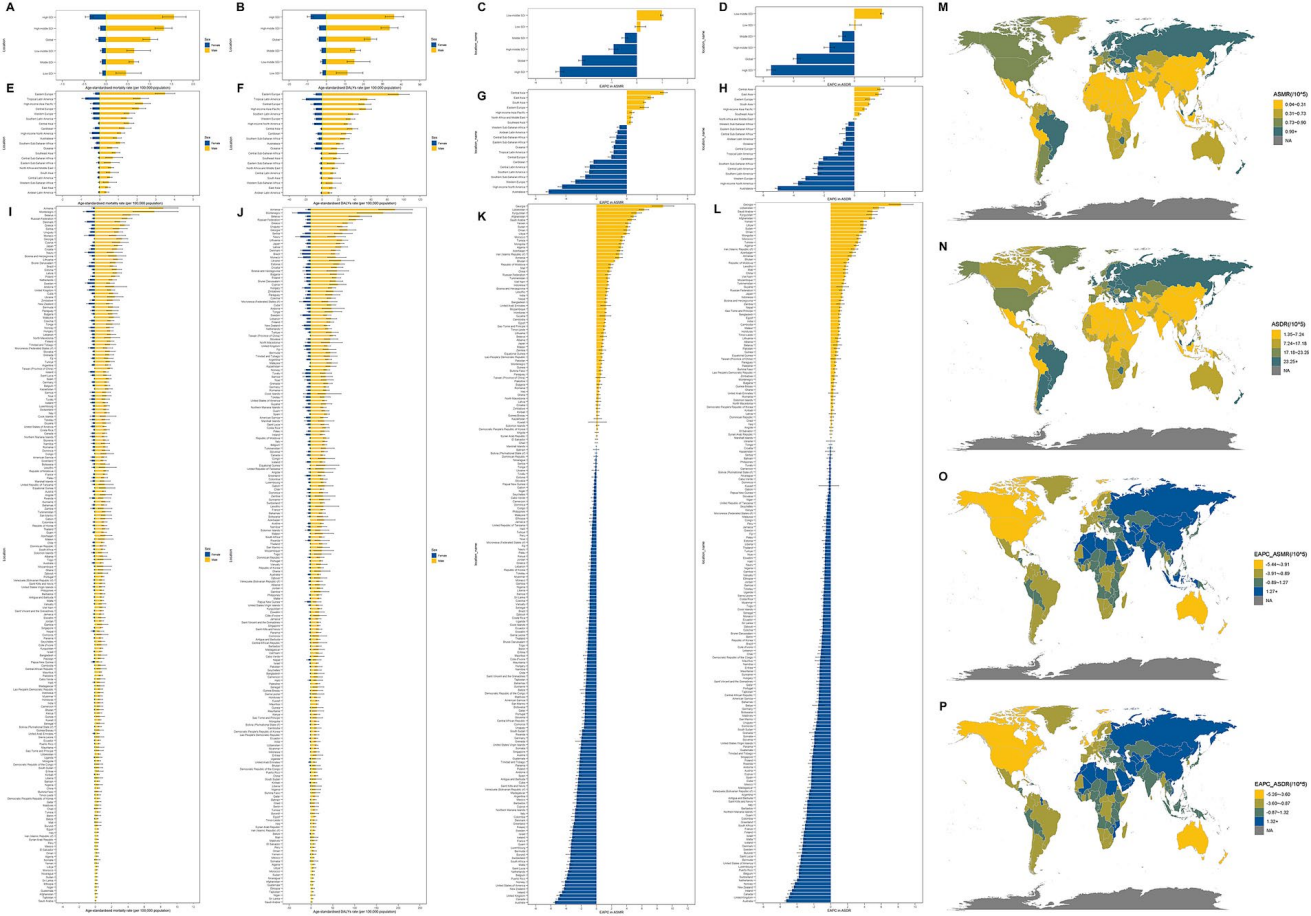
Temporal and spatial distribution of AA burden attributable to smoking in 204 countries and territories. **(A)** ASMRs and **(B)** ASDRs of AA attributable to smoking by global and five SDI in 2021; **(C)** EAPC in the ASMRs and **(D)** EAPC in the ASDRs of AA attributable to smoking by global and five SDI from 1990 to 2021; **(E)** ASMRs and **(F)** ASDRs of AA attributable to smoking by 21 regions in 2021; **(G)** EAPC in the ASMRs and **(H)** EAPC in the ASDRs of AA attributable to smoking by 21 regions from 1990 to 2021; **(I)** ASMRs and **(J)** ASDRs of AA attributable to smoking by 204 countries and territories in 2021; **(K)** EAPC in the ASMRs and **(L)** EAPC in the ASDRs of AA attributable to smoking by 204 countries and territories in 2021; **(M)** The global distribution of ASMRs and **(N)** ASDRs of AA attributable to smoking in 2021; **(O)** The global distribution of EAPCs in the ASMRs and **(P)** ASDR of AA attributable to smoking by country from 1990 to 2021.

In 2021, significant disparities in the smoking-attributable AA burden were observed across the global and 5 SDI region (Figures 3A–D). Simultaneously, significant disparities in the smoking-attributable AA burden were observed across 21 Global Burden of Disease (28) regions (Figures 3E–H). Eastern Europe emerged as the region with the highest ASMR and ASDR, recording 1.50 deaths (Figure 3E) and 43.68 DALYs (Figure 3F) per 100,000 population, respectively (Table 1). These figures starkly contrast with Andean Latin America, which reported the lowest burden at 0.2 ASMR (Figure 3E) and 5.01 ASDR (Figure 3F) per 100,000 population (Table 1). Notably, Central Asia experienced the most pronounced increase in both metrics, with estimated annual percentage changes (EAPCs) of 2.44 (95% UI: 2.22–2.66) for ASMR and 1.73 (95% UI: 1.53–1.94) for ASDR, highlighting accelerating regional challenges (Figures 3G,H). Conversely, Australasia demonstrated the steepest declines globally, achieving EAPCs of −5.14 (95% UI: −5.31 to −4.98) for ASMR and −5.04 (95% UI: −5.21 to −4.87) for ASDR, underscoring successful public health interventions in high-income settings (Table 1 and Figures 3G,H).

At the national level, Armenia, Montenegro, and Belarus led in ASMR (Figure 3I), while Armenia, Montenegro, and Belarus (Figure 3J) ranked highest for ASDR, reflecting persistent burdens in Eastern European and Caucasus countries (Supplementary Table S3). In contrast, Saudi Arabia, Tajikistan, and Afghanistan reported the lowest ASMR (Figure 3I), with Saudi Arabia, Sri Lanka, and Niger showing minimal ASDR (Figure 3J and Supplementary Table S4). Strikingly, Georgia, Uzbekistan, and Kyrgyzstan exhibited the fastest-growing AA burden with ASMR (Figure 3K), and Georgia, Uzbekistan, and Saudi Arabia exhibited the fastest-growing AA burden with ASDR (Figure 3L), EAPCs exceeding regional averages, suggesting underprioritized tobacco control in these areas (Supplementary Table S3). Meanwhile, Australia, the United Kingdom, and Canada achieved the most rapid reductions in both metrics, likely attributable to comprehensive anti-smoking policies and advanced healthcare systems (Figures 3K,L and Supplementary Table S4). Spatial distribution of AA burden attributable to smoking in 204 countries and territories (Figures 3M–P). The juxtaposition of Saudi Arabia having the lowest age-standardized mortality and disability rates yet exhibiting the fastest-growing burden of AA-related disability highlights a critical disconnect between current health outcomes and emerging risk factors, likely reflecting delayed consequences of insufficient preventive measures like tobacco control.

### 3.4 Age-period-cohort analysis uncovers declining global burden of smoking-attributable aortic aneurysm amid persistent age-specific risks

**Figure 4:**
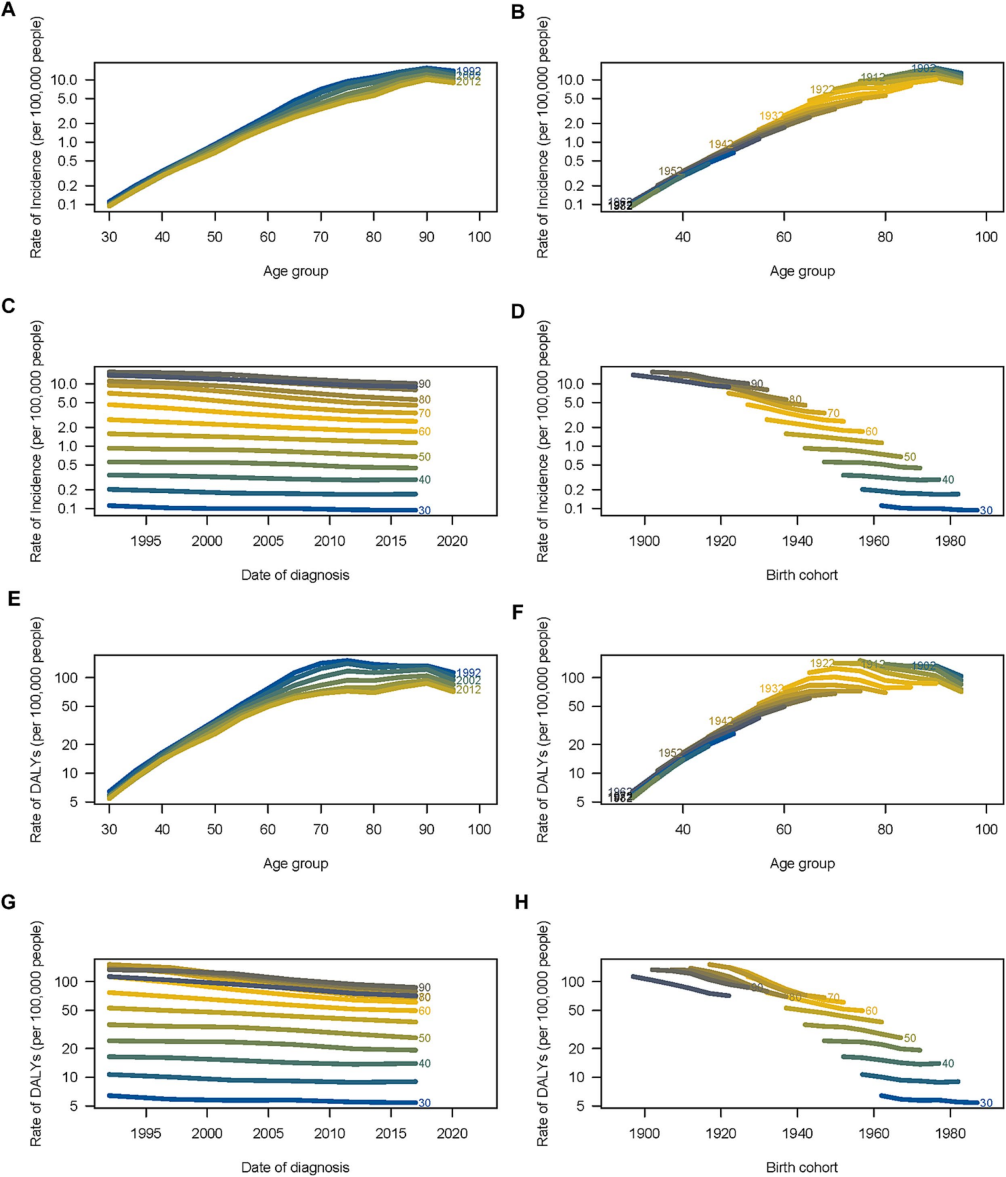
Age-period-cohort analysis mortality rates and DALYs of burden of smoking-attributable AA globally. The age-specific mortality rates of AA according to **(A)** time periods and **(B)** birth cohorts each line connects the age-specific mortality for a 5-year period; **(C)** The period-specific and **(D)** the birth cohort-specific mortality rates of AA according to age group, each line connects the birth cohort-specific mortality for a 5-year age group; The age-specific DALY rates of AA according to **(E)** time periods and **(F)** birth cohorts; each line connects the age-specific DALY for a 5-year period; **(G)** The period-specific and **(H)** the birth cohort DALY rates of AA according to age group, each line connects the birth cohort-specific DALY for a 5-year age group.

Longitudinal trends in mortality rates and DALYs associated with smoking-attributable AA revealed distinct age-dependent patterns over three decades. The age-stratified analysis (30–100 years) reveals a nonlinear increase in mortality (Figure 4A) and DALY rates (Figure 4E) with advancing age, peaking at approximately 60 per 100,000 in individuals aged 80–100. Curves for consecutive years (1992–2012) demonstrate a progressive upward shift in the age gradient, suggesting cumulative smoking-related vascular damage over the lifespan. The highest burden occurs in older populations, aligning with age-dependent biological vulnerability. Cohorts born 1902 exhibit the highest rates (up to10 per 100,000) for mortality (Figure 4B), and cohorts born between 1902 and 1922 exhibit the highest rates (up to 1,000 per 100,000) for DALYs (Figure 4F) reflecting prolonged exposure to smoking during periods of high tobacco prevalence. A gradual decline in post-1932 cohorts coincide with down-regulation of smoking initiation rates and public health interventions in later decades, highlighting generational shifts in risk. Temporal trends stratified by age group (Figures 4C,G) highlighted a modest but consistent decline in both DALYs and mortality across all ages between 1990 and 2017, with the most pronounced improvements observed in populations aged 50–70. However, older adults (≥80 years) maintained disproportionately higher rates, underscoring the cumulative impact of smoking exposure and age-related vascular vulnerability. The parallel downward trajectories across age groups suggest systematic improvements in prevention, diagnosis, or treatment (e.g., smoking cessation programs, surgical advancements). Cohort-effect analyses (Figures 4D,H) revealed declining mortality ratios in successive birth cohorts, particularly after 1950, likely reflecting reduced smoking prevalence and advancements in cardiovascular care. Notably, data limitations precluded analysis of the 0–30 age group, leaving gaps in understanding early-life risk trajectories. A marked reduction in DALY rates is observed across successive birth cohorts. Early cohorts (1900–1950) exhibit rates as high as 70 per 100,000, likely due to limited awareness of smoking risks and delayed clinical management. In contrast, cohorts born post-1950 show a progressive decline (to 30 per 100,000), underscoring the impact of anti-smoking policies and earlier medical interventions.

### 3.5 Decomposition analysis of smoking-related AA burden: key drivers and regional variations

**Figure 5:**
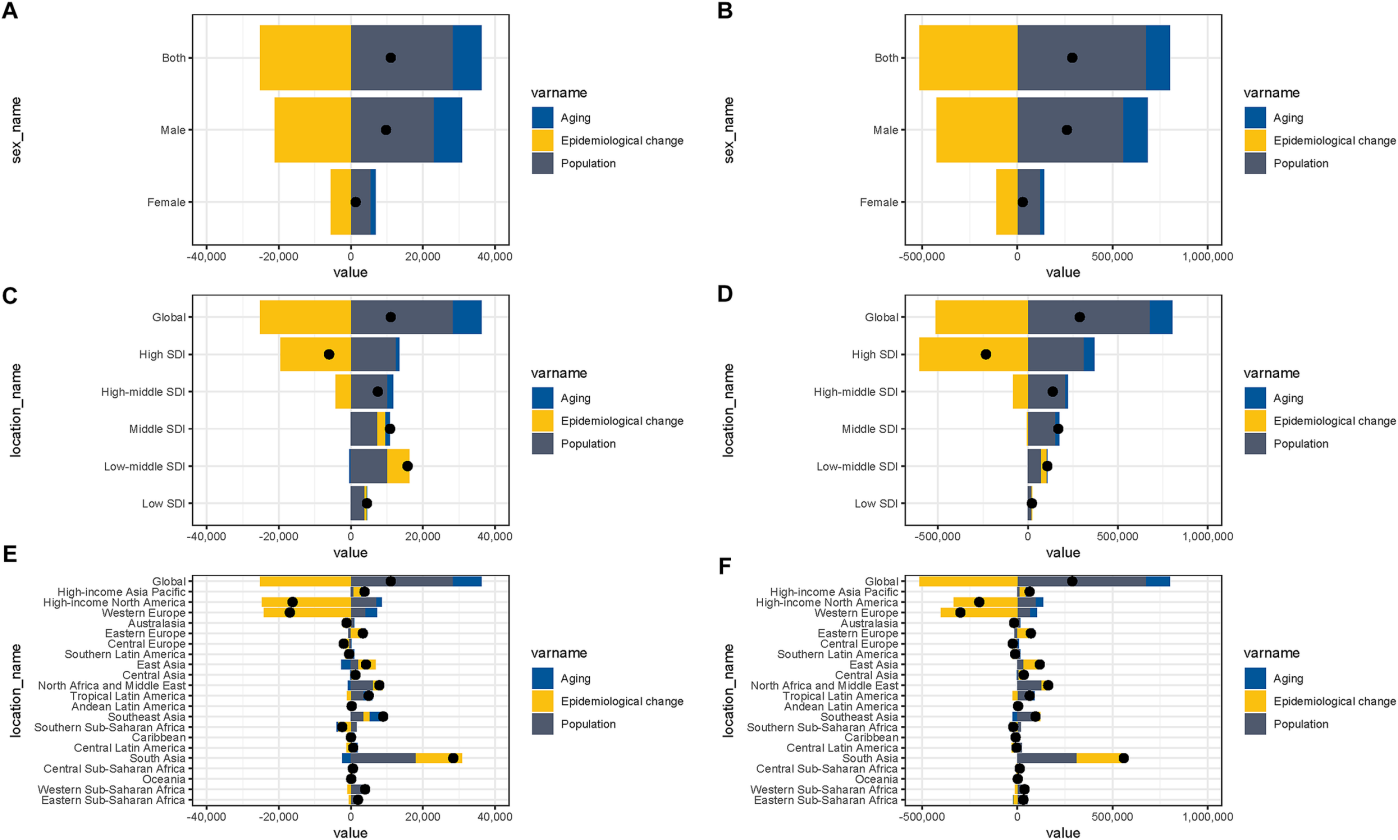
Decomposition analysis of mortality and DALYs and due to AA attributable to smoking worldwide, 1990–2021. Changes in both sexual, male and female **(A)** mortality and **(B)** DALYs for AA attributable to smoking; Changes in global and sociodemographic index (SDI) quintile-specific **(C)** mortality rates and **(D)** DALYs for AA attributable to smoking; Changes in global and 21 regions **(E)** mortality rates and **(F)** DALYs for AA attributable to smoking.

A decomposition analysis was conducted to disentangle the contributions of aging, demographic expansion, and shifts in disease epidemiology to smoking-attributable AA burden, as measured through both mortality DALYs. Globally, between 1990 and 2021, demographic growth emerged as the dominant driver of rising mortality for AA linked to smoking, accounting for 255.44% of the observed increase. This was partially offset by epidemiological changes (−227.57%), which reflected improvements in prevention, diagnosis, or treatment, while population aging contributed an additional 72.13% to the burden (Supplementary Table S5 and Figure 5A). A parallel pattern was observed for DALYs (Supplementary Table S6 and Figure 5B), where population growth (234.19%) and aging (44.28%) synergistically elevated deaths, though these effects were counterbalanced by epidemiological advancements (−178.46%). Sex-stratified analysis revealed that population growth disproportionately amplified the burden for both males and females worldwide. However, nuanced gender-specific variations in aging rates and epidemiological exposures (e.g., differential smoking cessation trends or occupational risks) may underlie divergences in regional outcomes, warranting further investigation. These findings underscore the critical interplay between demographic dynamics and public health interventions in shaping the global burden of smoking-associated diseases (Figures 5A,B). Regionally, low to high-middle SDI areas exhibited consistent trends, with demographic expansion serving as the primary catalyst for escalating DALYs and mortality. In contrast, high SDI regions demonstrated a reversal of this trajectory, where progressive epidemiological shifts—potentially linked to tobacco control policies, reduced smoking prevalence, or enhanced healthcare access—substantially mitigated AA-related health losses (Figures 5C–F).

### 3.6 Socioeconomic development and smoking-attributable AA burden: threshold dynamics and regional disparities

**Figure 6:**
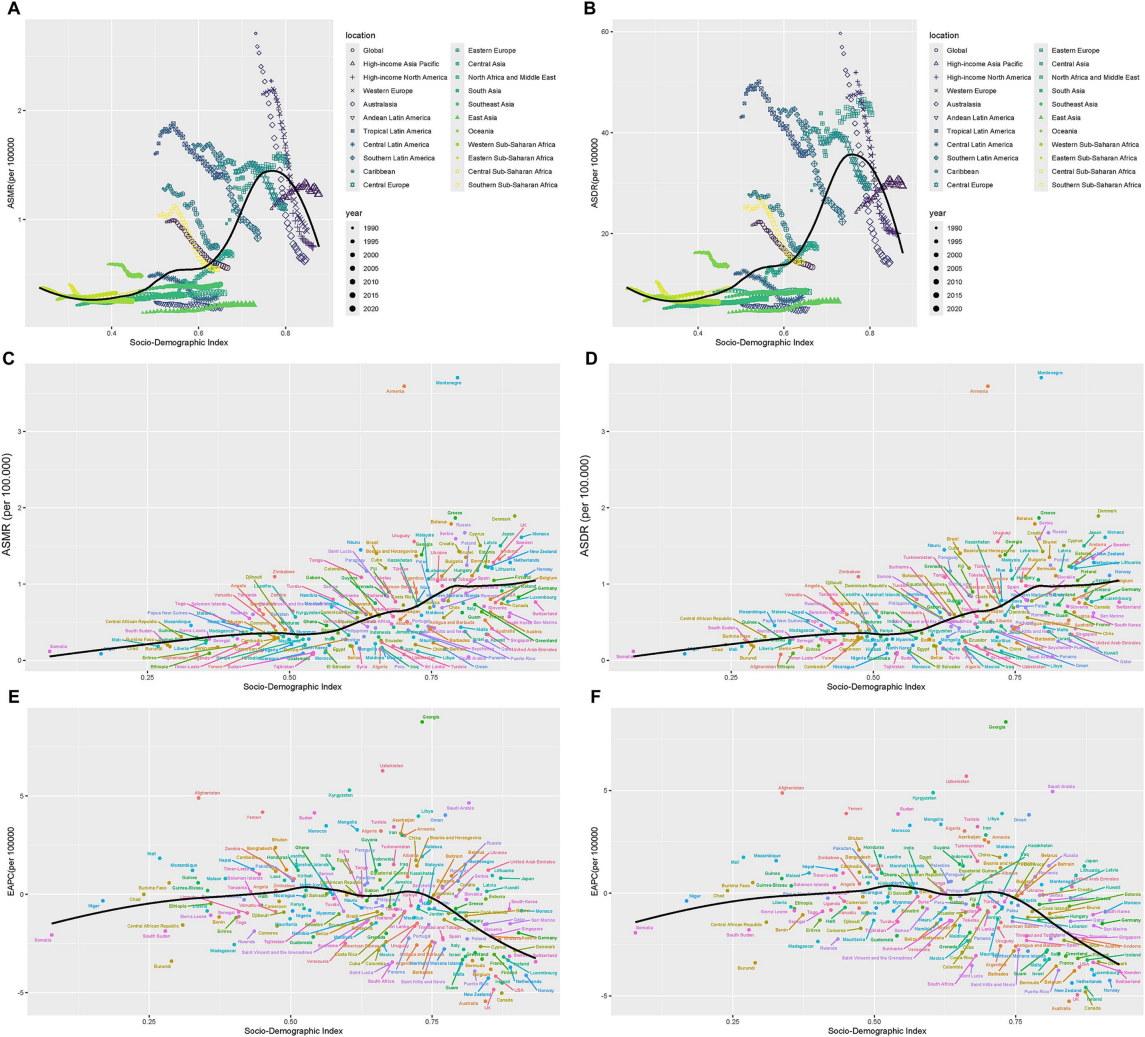
The correlation between SDI and burden of AA attributable to smoking. The correlation between SDI and **(A)** ASMR and **(B)** ASDR of AA attributable to smoking in 21 GBD regions in 2021; The correlation between SDI and **(C)** ASMR and **(D)** ASDR of AA attributable to smoking in 204 GBD countries in 2021; The correlation between SDI and **(E)** EAPC for ASMR and **(F)** EAPC for ASDR of AA attributable to smoking in 204 GBD countries in 2021. The SDI threshold (~0.75) marking the transition from positive to negative associations was identified through visual inspection and LOESS regression.

Between 1990 and 2021, the age-standardized mortality rate (Figure 6A) and age-standardized disability-adjusted life year (Figure 6B) and for smoking-related AA exhibited a nonlinear association with socioeconomic development, as quantified by SDI across 21 GBD regions (28). While both ASDR and ASMR initially rose in tandem with increasing SDI, a distinct reversal occurred at higher SDI levels (above 0.75), where the burden declined sharply. Socioeconomic analysis revealed a nonlinear threshold pattern: AA burden increased with SDI up to approximately 0.75 (empirically identified via LOESS regression), beyond which further development correlated with declining burden. This suggests that effective health systems and tobacco control policies may counteract AA risks in high-SDI settings. This pattern suggests a development threshold effect: below an SDI of 0.75, socioeconomic progress correlated with elevated AA burden, likely driven by lifestyle shifts, increased tobacco accessibility, or delayed implementation of preventive measures. However, beyond this threshold, enhanced healthcare infrastructure, stricter tobacco regulations, and public health awareness campaigns appeared to counteract these risks, driving down AA-related morbidity and mortality. Regional disparities further underscored this relationship. For instance, Eastern Europe and Tropical Latin America exhibited AA burdens exceeding model predictions, potentially reflecting persistent high smoking prevalence, weaker tobacco control enforcement, or occupational exposures in these regions. Conversely, East Asia, Southeast Asia, and Western Europe demonstrated lower-than-expected burdens, possibly attributable to early adoption of smoking cessation programs, culturally influenced reductions in tobacco use, or advancements in early disease detection and management. For instance, as High-Income Asia Pacific, with an SDI > 0.85, Japan aligns with the downward trend post-SDI 0.75, reflecting low ASMR (e.g., ~1.5 per 100,000) and ASDR (e.g., ~1.5 per 100,000) due to stringent tobacco taxes, widespread smoking cessation programs, and advanced aortic disease screening. For Montenegro (Central Europe), despite moderate SDI (~0.75), Montenegro’s ASMR exceeds predictions (e.g., ~3.5 per 100,000), driven by historically high male smoking rates (>30%) and delayed implementation of EU tobacco directives. And for Brazil (Tropical Latin America) with SDI ~ 0.70, Brazil’s ASMR remains elevated (~1.5 per 100,000), potentially linked to persistent rural smoking cultures and uneven healthcare access despite national anti-smoking campaigns (Figures 6C–F).

### 3.7 Projected burden of smoking-attributable aortic aneurysm: gender disparities and global trends (2022–2036)

**Figure 7:**
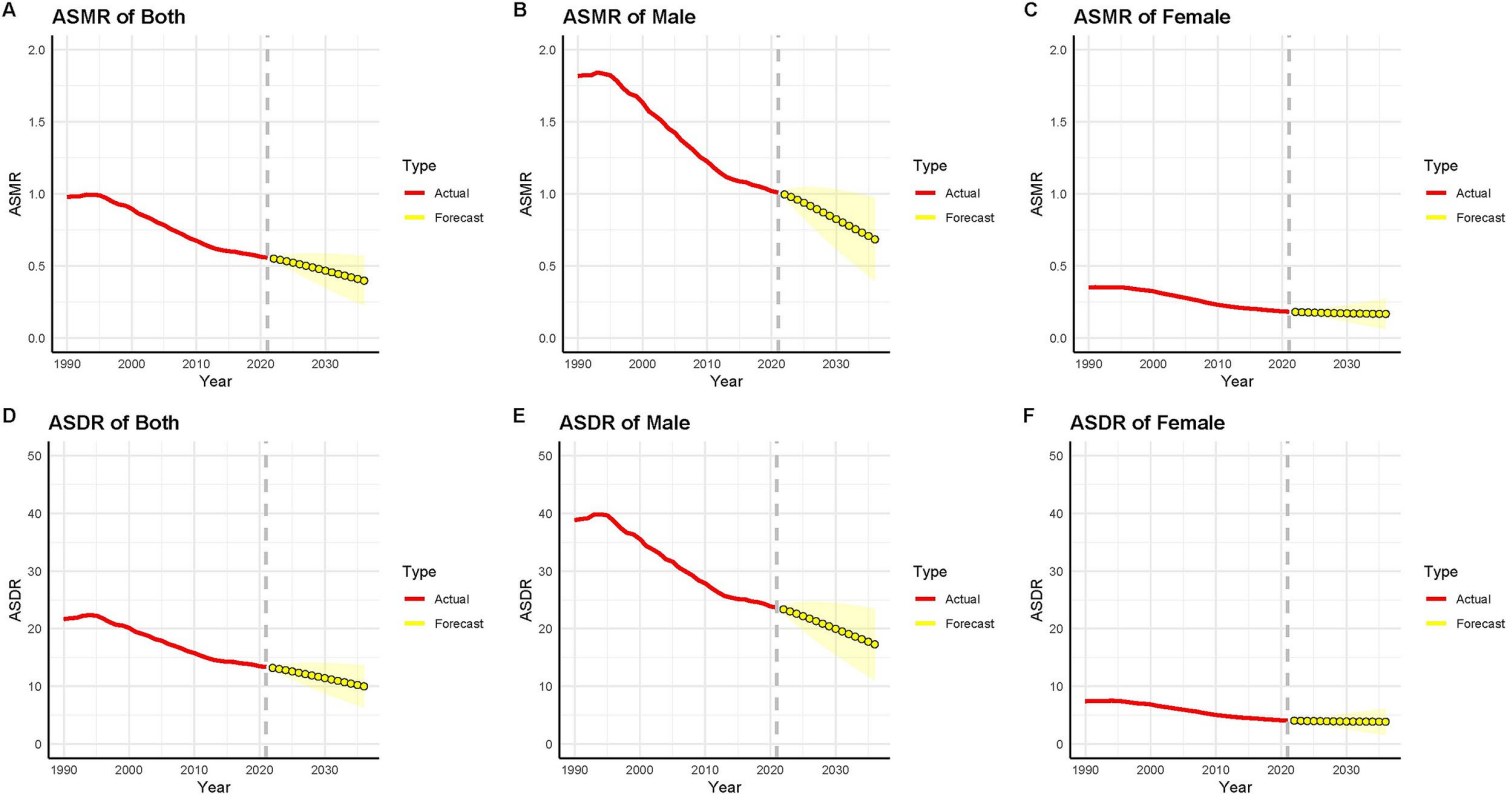
Prediction of ASMR and ASDR of AA from 2022 to 2036 caused by smoking global. Prediction of AAs ASMR caused by smoking by **(A)** both genders, **(B)** males, and **(C)** females in global; Prediction of AAs ASDR caused by smoking by **(D)** both genders, **(E)** males, and **(F)** females in global. Dashed lines represent 95% prediction intervals. Projections assume stable historical trends in smoking exposure and AA management.

Globally, ASMR and ASDR for smoking-related AA are projected to decline steadily between 2022 and 2036 (Figures 7A,D). By 2036, the ASMR is expected to drop to 0.40 per 100,000 population, a 28.6% reduction from 0.56 per 100,000 in 2021 (Figure 7A), while ASDR is forecasted to decrease by 25.1%, reaching 9.99 per 100,000 compared to 13.35 per 100,000 in 2021 (Figure 7D). These trends signal progress in mitigating smoking-related health burdens, likely driven by expanded tobacco control policies, advancements in disease management, and shifts in population health behaviors. Gender-stratified projections reveal persistent disparities. Males are anticipated to experience higher ASMR and ASDR than females throughout the period (Figures 7B,C,E,F, respectively). By 2036, male ASMR and ASDR are estimated at 0.68 and 17.3 per 100,000, respectively, reflecting reductions of 32.3 and 27.0% from 2021 levels (Figures 7B,E). In contrast, female rates are projected to decline more modestly—8.5% for ASMR (0.17 per 100,000, Figure 7C) and 5.2% for ASDR (3.85 per 100,000, Figure 7F). This divergence may stem from historically higher smoking prevalence among males, slower cessation rates in certain male populations, or occupational exposures amplifying risks. However, the slower decline in female burdens could signal emerging public health challenges, such as rising smoking initiation among women in specific regions or biological susceptibility differences. Notably, while global trends indicate overall improvement, regional and demographic heterogeneities may obscure localized increases. For instance, populations with stagnating tobacco control measures or inadequate healthcare access might defy these projections. Furthermore, the relatively modest reductions in female burdens underscore the need for gender-sensitive interventions, such as targeted anti-smoking campaigns addressing sociocultural norms or pregnancy-related risks. These forecasts highlight both the successes of global health initiatives and the urgency of addressing persistent inequities in smoking-attributable disease prevention.

### 3.8 Geographic disparities in smoking-attributable AA mortality and DALYs rates by region in 2021

**Figure 8:**
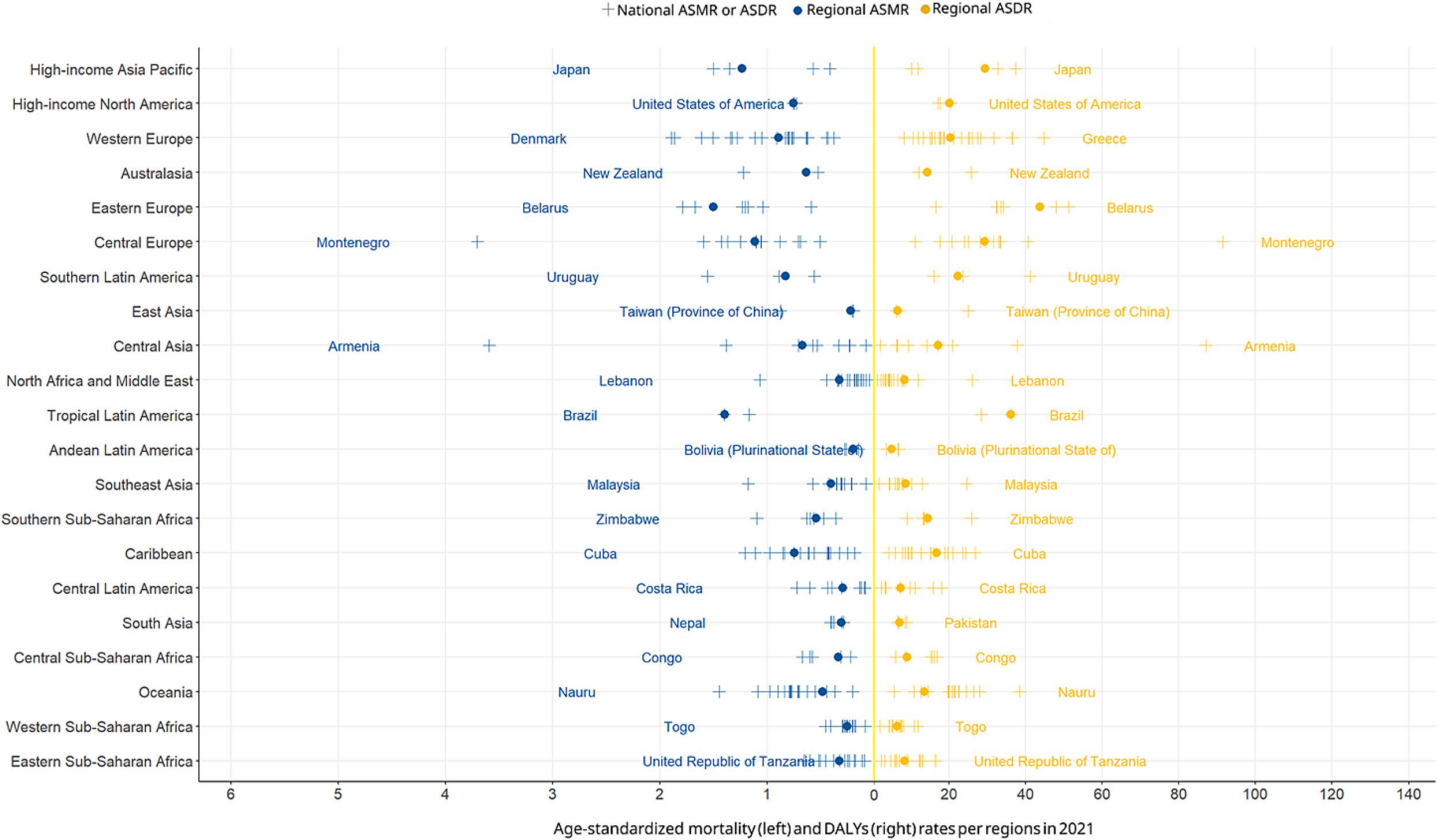
Geographic disparities in smoking-attributable AA mortality and DALYs rates by region in 2021.

As shown in Figure 8, it illustrates the geographic heterogeneity in ASMR and disability-adjusted life years rates attributable to smoking-related aortic aneurysm across 204 countries and 21 GBD regions in 2021. Each vertical strip represents a GBD region, with blue circles indicating the region’s average ASMR and yellow circles indicating the region’s average ASDR, and “+” symbols marking the country with the highest rate (ASMR or ASDR) within that region. Notably, Eastern Europe (Belarus) exhibited the highest national ASMR (1.5, 95%UI: 1.28–1.72) and ASDR (43.68, 95%UI: 37.66–49.23). Similarly, Tropical Latin America (Brazil) recorded the highest ASMR (1.4, 95%UI: 1.16–1.68) and ASDR (36.07, 95%UI: 30.7–42.22) in its region. Striking outliers emerged in East Asia, where TAIWAN PROVINCE OF CHINA ASMR is 0.22 (95%UI: 0.16–0.28) and ASDR is 6.6 (95%UI: 4.84–8.58) represented the highest national rates, despite this region’s relatively low averages ASMR and ASDR. This spatial stratification highlights systemic inequities: low-resource regions (e.g., Oceania, South Asia) exhibited both elevated regional averages and extreme national outliers, whereas high-income regions maintained lower and more homogeneous rates. The bidirectional divergence underscores how socioeconomic development and tobacco control policies modulate smoking-attributable AA burdens at subregional levels.

## 4 Discussion

The present study offers a comprehensive evaluation of the global, regional, and national burden of AA attributable to smoking based on the Global Burden of Disease 2021 data. GBD data were selected as the sole source because its standardized estimation framework (disability weights, comorbidity adjustments, crosswalking) enables valid comparisons across time/regions-unachievable by combining local registries with divergent diagnostic criteria, coding practices, and coverage. While model-based estimates carry uncertainty, GBD’s systematic data processing minimizes bias in trend analyses. Our findings indicate that while age-standardized mortality and DALYs rates attributable to smoking have generally declined since 1990, the absolute number of smoking-attributable deaths and DALYs due to AA continues to rise globally. This discrepancy can largely be attributed to population growth and demographic aging. The burden is disproportionately higher among males and older adults, and significant regional disparities exist, with high-income regions such as North America and parts of Europe bearing the highest burden in absolute terms. Conversely, several low- and middle-SDI countries are seeing increasing trends, likely reflecting rising smoking prevalence and limited healthcare infrastructure. A strong correlation between the SDI and the burden suggests that both development level and health systems capacity play an important role in AA epidemiology.

The strong association between smoking and AA aligns with known pathophysiological mechanisms, might including elastin degradation and extracellular matrix remodeling, vascular inflammation and oxidative stress, smooth muscle cell apoptosis, hemodynamic stress, and endothelial dysfunction. First, smoking activates matrix metalloproteinases (MMP), notably MMP-2 and MMP-9, which degrade elastin and collagen in the aortic wall. Research showing that cigarette smoke extract induces expression of MMP-2 as well as MMP-9 and that nicotine, through increased expression of MMP-2 induces AAA (29, 30). Intriguingly, the influence of smoking on aneurysm progression persists despite the administration of pharmacological protease inhibitors or the application of genetic knockout strategies targeting elastase-producing enzymes (31). Notably, researchers propose that the impact of smoking on AAA progression exhibits greater severity in female populations. Their analysis highlights that woman demonstrate higher motivation to cease smoking yet encounter greater challenges in achieving cessation compared to male counterparts, a behavioral contrast that may underlie the observed increased aggressiveness of AAA pathogenesis in this demographic (32). Moreover, experimental models demonstrate that cigarette smoke condensate induces programmed cell death across diverse vascular cell lineages, including aortic smooth muscle cells (SMCs), human umbilical vein endothelial cells (33), and pulmonary/aortic endothelial cells derived from multiple species (34). However, mechanistic studies paradoxically reveal nicotine, the primary bioactive component of cigarette smoke, suppresses SMC and endothelial cell apoptosis via nicotinic acetylcholine receptor-mediated pathways in multiple experimental systems (35). Current empirical data ultimately contradict the hypothesis implicating nicotine-mediated apoptotic mechanisms in either initiation or pathological evolution of AAAs (36). Finally, impaired nitric oxide bioavailability in smokers contributes to pro-inflammatory and pro-thrombotic vascular environments. These mechanisms collectively explain the significant and often irreversible damage caused by long-term smoking on aortic structure (37).

While age-standardized rates (ASRs) of AA burden attributable to smoking have declined in most regions, the absolute burden continues to grow. This paradox is explained as followed. First, older age is a major risk factor for AA; thus, an aging demographic magnifies the absolute burden (38). Secondly, as countries shift from infectious to chronic disease patterns, non-communicable diseases like AA become more prominent (28). In some regions, better access to imaging has increased AA detection rates, especially for asymptomatic cases. In this study, we found that high-SDI countries generally show declining trends in smoking-might attributable AA burden, which due to long-standing tobacco control policies, high healthcare capacity for early detection and management, and public awareness campaigns (39). High-SDI countries have observed declines in AA burden, likely due to effective tobacco control and hypertension screening programs, many low-SDI countries continue to face barriers in policy implementation and healthcare access. For instance, smoking cessation policies, though adopted in many regions, often suffer from weak enforcement and lack of culturally adapted intervention programs. In contrast, many low- and middle-SDI countries are witnessing increasing AA burden due to rising smoking prevalence, especially among young men and women, limited access to screening and surgical treatment, and aggressive tobacco marketing and policy inertia (40, 41). The increasing AA burden in low- and middle-SDI regions may reflect both epidemiological transitions and incomplete implementation of primary prevention strategies. Pathophysiologically, AA is strongly linked to chronic inflammation and degradation of vascular wall structural proteins, processes that are accelerated by long-term smoking exposure and uncontrolled hypertension. Our findings suggest that greater investment in tobacco control infrastructure, along with expansion of blood pressure monitoring and treatment services, should be prioritized in low-SDI regions. Moreover, targeted public health campaigns and surveillance systems are crucial for early detection and intervention. This SDI-based disparity highlights the importance of global equity in NCD prevention.

Consistent with previous studies, our analysis confirms that males constitute the majority of the smoking-attributable AA burden, likely due to higher lifetime smoking exposure, sex-specific vascular susceptibility, and behavioral or cultural factors. However, we also observed a notable upward trend in the AA burden among females, particularly in upper-middle SDI countries (42). This trend may reflect increasing tobacco use among women, under-recognition of AA in females due to atypical clinical presentations and less frequent screening, as well as a higher risk of aneurysm rupture at smaller diameters compared to males (43). These findings underscore the importance of implementing sex-specific screening protocols and developing targeted public health messaging to address the unique risk profile of women. Compared with prior research, our study contributes greater global granularity by explicitly quantifying the smoking-attributable AA burden across different regions, sexes, and levels of sociodemographic development. For instance, Wang et al. (13), Jing et al. (14), and Tian et al. (15), highlighting both similarities and differences. While all these studies report general trends such as a decline in aortic aneurysm (AA) mortality in high-SDI regions, increasing trends in low-SDI regions, and male predominance, our study offers distinct contributions. First, unlike Wang et al., which focuses on early-onset AA and projections to 2045, we examine the smoking-attributable AA burden across all age groups, with projections extending only to 2036 (13). While Jing et al. explores the impact of BMI on AA burden, our study isolates the specific burden attributable to smoking, employing a combination of Joinpoint, APC models, decomposition analysis, and ARIMA forecasting for a multidimensional assessment (14). Furthermore, our forecasting model incorporates gender-specific trajectories, an approach not used in Tian et al., which focuses on overall AA burden. The methodological depth and the specific focus on smoking-attributable AA burden are the primary novelties in our study, providing new insights into the potential public health burden of smoking-related AA mortality, despite confirming trends seen in prior research. By isolating smoking-related mortality and applying advanced statistical modeling techniques, our study offers a more targeted approach for future health policies and interventions.

To effectively reduce the global burden of smoking-attributable AA, it is crucial to strengthen tobacco control measures, particularly in low- and middle-income countries. This study provides the first comprehensive assessment of smoking-attributable AA burden, identifying a development-dependent threshold effect that redefines global tobacco control priorities. This can be achieved by enforcing stricter tobacco taxation, advertising bans, plain packaging, and smoke-free laws. In parallel, expanding AA screening in high-risk populations, such as men aged 65 + with a history of smoking, should be prioritized, with consideration for extending these screening efforts to women who present similar risk profiles. Integrating smoking cessation programs into national non-communicable disease strategies, particularly within cardiovascular care frameworks, is essential for reducing the smoking-related AA burden. Furthermore, health system capacity in LMICs needs to be bolstered through investments in imaging technologies, surgical services, and healthcare workforce training. Public health campaigns should also be tailored to emphasize the link between smoking and aortic diseases—not just focusing on cancers. Despite the strengths of using the comprehensive GBD 2021 dataset, which provides extensive coverage across regions and decades, our study is not without limitations. The ecological design of the study limits inferences at the individual level, and the lack of data on AA subtypes (thoracic vs. abdominal) restricts our ability to analyze their potentially distinct risk profiles. Although Joinpoint regression, the age-period-cohort (APC) model, decomposition analysis, and ARIMA forecasting methods are commonly used, they have limitations. For example, Joinpoint regression assumes a fixed number of segments, which may not always align with the true data structure. The APC model, while powerful, can be sensitive to model assumptions and the choice of age groups. Decomposition analysis may oversimplify complex causal relationships, and ARIMA forecasting assumes linearity in the data, which may not capture all non-linear patterns. We have considered these limitations in our analysis and believe that these methods provide a robust framework for examining the research questions at hand. Additionally, the population-level smoking data do not capture factors such as intensity, duration, or cessation patterns, and data modeling in LMICs may be affected by underreporting or sparse vital statistics. They extrapolate past trends without modeling structural shifts in tobacco markets (e.g., vaping adoption) or healthcare systems. Policymakers should treat these as baseline scenarios requiring continuous reassessment against real-world surveillance data, particularly in low-SDI regions where our models show highest volatility. Nevertheless, our research represents one of the most detailed global assessments of the smoking-attributable burden of AA to date.

Looking ahead, our findings underscore the critical need for targeted public health strategies that directly address smoking-related aortic aneurysm (AA) burden. Future research should aim to validate our projections through real-world longitudinal studies, particularly in low- and middle-income countries where data gaps remain significant. Moreover, integrating individual-level behavioral data, such as smoking cessation patterns, second-hand smoke exposure, and comorbidity profiles, could refine current models and strengthen causal inference. From a methodological standpoint, combining machine learning-based forecasting with traditional epidemiological models may offer more robust predictive insights. Lastly, exploring interactions between smoking and emerging risk factors like air pollution or genetic susceptibility could open new avenues for prevention and policy development.

## 5 Conclusion

Our study reveals that while age-standardized mortality and disability rates of AA attributable to smoking have declined globally from 1990 to 2021, the absolute burden continues to rise due to population growth and aging. Striking disparities persist across regions, genders, and socioeconomic levels, with low- and middle-SDI countries experiencing growing burdens, in contrast to high-SDI regions that show significant declines. The age-period-cohort and decomposition analyses highlight those older adults bear the highest burden, primarily driven by demographic shifts, while epidemiological improvements have had a mitigating effect. Importantly, our forecasts suggest continued global declines through 2036, yet without targeted interventions, inequities are likely to persist. Importantly, the sex-specific forecasting reveals different risk trajectories, which could guide more personalized and equitable public health interventions. Our findings underscore the urgency of implementing gender-specific screening protocols and public health messaging addressing these compounded vulnerabilities. These insights are directly applicable to clinical practice and health policy, particularly in strengthening smoking cessation programs and optimizing screening strategies for at-risk populations. The originality of this work lies in its focused estimation of smoking-attributable burden, providing a critical lens for more targeted and actionable vascular health interventions in the coming decade. These findings underscore the need for enhanced tobacco control policies and equitable health system strengthening to address smoking-related AA burden worldwide.

## Data Availability

The original contributions presented in the study are included in the article/Supplementary material, further inquiries can be directed to the corresponding authors.

## Footnotes

^1^ https://ghdx.healthdata.org/gbd-2021

## References

[1] Goyal A Saeed H Shahnoor S Arshad MK Wasay A Abdullah. Mortality trends, sex, and racial disparities in older adults due to abdominal aortic aneurysm: a nationwide cross-sectional analysis. Int J Surg. (2024) 110:8241–5. doi: 10.1097/JS9.0000000000002114 39806755 PMC11634149

[2] Wang Z You Y Yin Z Bao Q Lei S Yu J. Burden of aortic aneurysm and its attributable risk factors from 1990 to 2019: an analysis of the global burden of disease study 2019. Front Cardiovasc Med. (2022) 9:901225. doi: 10.3389/fcvm.2022.901225, PMID: 35711350 PMC9197430

[3] Forsdahl SH Singh K Solberg S Jacobsen BK. Risk factors for abdominal aortic aneurysms: a 7-year prospective study: the Tromso study, 1994-2001. Circulation. (2009) 119:2202–8. doi: 10.1161/CIRCULATIONAHA.108.817619 19364978

[4] Abdulameer H Al Taii H Al-Kindi SG Milner R. Epidemiology of fatal ruptured aortic aneurysms in the United States (1999-2016). J Vasc Surg. (2019) 69:378–384.e2. doi: 10.1016/j.jvs.2018.03.435, PMID: 29960790

[5] Yan L Dexin S Ge Z Bo W Haiyu W Jinying Z. Causal associations between the gut microbiome and aortic aneurysm: a mendelian randomization study. CVIA. (2024) 9:23. doi: 10.15212/CVIA.2024.0023

[6] Norman PE Curci JA. Understanding the effects of tobacco smoke on the pathogenesis of aortic aneurysm. Arterioscler Thromb Vasc Biol. (2013) 33:1473–7. doi: 10.1161/ATVBAHA.112.300158, PMID: 23685557 PMC3683352

[7] Song K Guo C Yang K Li C Ding N. Clinical characteristics of aortic aneurysm in MIMIC-III. Heart Surg Forum. (2021) 24:E351–8. doi: 10.1532/hsf.3571, PMID: 33798047

[8] Shen YH LeMaire SA. Molecular pathogenesis of genetic and sporadic aortic aneurysms and dissections. Curr Probl Surg. (2017) 54:95–155. doi: 10.1067/j.cpsurg.2017.01.001, PMID: 28521856 PMC7335366

[9] Liu B Granville DJ Golledge J Kassiri Z. Pathogenic mechanisms and the potential of drug therapies for aortic aneurysm. Am J Physiol Heart Circ Physiol. (2020) 318:H652–70. doi: 10.1152/ajpheart.00621.2019, PMID: 32083977 PMC7099451

[10] Fu M Mei A Min X Yang H Wu W Zhong J. Advancements in cardiovascular disease research affected by smoking. Rev Cardiovasc Med. (2024) 25:298. doi: 10.31083/j.rcm2508298, PMID: 39228476 PMC11367002

[11] Khan Minhas AM Sedhom R Jean ED Shapiro MD Panza JA Alam M. Global burden of cardiovascular disease attributable to smoking, 1990-2019: an analysis of the 2019 global burden of disease study. Eur J Prev Cardiol. (2024) 31:1123–31. doi: 10.1093/eurjpc/zwae040, PMID: 38589018

[12] Sweeting MJ Thompson SG Brown LC Powell JT collaborators R. Meta-analysis of individual patient data to examine factors affecting growth and rupture of small abdominal aortic aneurysms. Br J Surg. (2012) 99:655–65. doi: 10.1002/bjs.8707 22389113

[13] Wang H Li Y Fan K Zhao T Xu K Zahin M. Global epidemiology of early-onset aortic aneurysm: temporal trends, risk factors, and future burden projections. J Epidemiol Glob Health. (2025) 15:25. doi: 10.1007/s44197-025-00369-y, PMID: 39945980 PMC11825438

[14] Guo ZZ Cao QA Li ZZ Liu LP Zhang Z Zhu YJ. SP600125 attenuates nicotine-related aortic aneurysm formation by inhibiting matrix metalloproteinase production and CC chemokine-mediated macrophage migration. Mediat Inflamm. (2016) 2016:9142425. doi: 10.1155/2016/9142425 PMC5023844 27688602

[15] Tian C Zhang X Tang H Luo N Huang J Lin H. Disease burden of aortic aneurysm from 1990 to 2021 with a forecast to 2045: insights from the global burden of disease 2021. BMC Public Health. (2025) 25:1829. doi: 10.1186/s12889-025-23067-7, PMID: 40382603 PMC12085002

[16] Aune D Schlesinger S Norat T Riboli E. Tobacco smoking and the risk of abdominal aortic aneurysm: a systematic review and meta-analysis of prospective studies. Sci Rep. (2018) 8:14786. doi: 10.1038/s41598-018-32100-2, PMID: 30283044 PMC6170425

[17] Zhang F Kent KC Yamanouchi D Zhang Y Kato K Tsai S. Anti-receptor for advanced glycation end products therapies as novel treatment for abdominal aortic aneurysm. Ann Surg. (2009) 250:416–23. doi: 10.1097/SLA.0b013e3181b41a18, PMID: 19652591 PMC2921961

[18] Thompson A Fleischmann KE Smilowitz NR de Las Fuentes L Mukherjee D Aggarwal NR. AHA/ACC/ACS/ASNC/HRS/SCA/SCCT/SCMR/SVM guideline for perioperative cardiovascular management for noncardiac surgery: a report of the American College of Cardiology/American Heart Association joint committee on clinical practice guidelines. Circulation. (2024) 150:e351–442. doi: 10.1161/CIR.0000000000001285 39316661

[19] Feng G Qin G Zhang T Chen Z Zhao Y. Common statistical methods and reporting of results in medical research. CVIA. (2022) 6:117–25. doi: 10.15212/CVIA.2022.0001

[20] Yang JJ Trucco EM Buu A. A hybrid method of the sequential Monte Carlo and the Edgeworth expansion for computation of very small p-values in permutation tests. Stat Methods Med Res. (2019) 28:2937–51. doi: 10.1177/0962280218791918, PMID: 30073912 PMC6360137

[21] Ormiston CK Lawrence WR Sulley S Shiels MS Haozous EA Pichardo CM. Trends in adolescent suicide by method in the US, 1999-2020. JAMA Netw Open. (2024) 7:e244427. doi: 10.1001/jamanetworkopen.2024.4427, PMID: 38551558 PMC10980967

[22] Fu W Ding J Gao K Ma S Tian L. A likelihood ratio test on temporal trends in age-period-cohort models with applications to the disparities of heart disease mortality among US populations and comparison with Japan. Stat Med. (2021) 40:668–89. doi: 10.1002/sim.8796, PMID: 33210329 PMC10676755

[23] Osmond C Gardner MJ. Age, period and cohort models applied to cancer mortality rates. Stat Med. (1982) 1:245–59. doi: 10.1002/sim.4780010306, PMID: 7187097

[24] Holford TR. The estimation of age, period and cohort effects for vital rates. Biometrics. (1983) 39:311–24. PMID: 6626659

[25] Cheng X Tan L Gao Y Yang Y Schwebel DC Hu G. A new method to attribute differences in total deaths between groups to population size, age structure and age-specific mortality rate. PLoS One. (2019) 14:e0216613. doi: 10.1371/journal.pone.0216613, PMID: 31075117 PMC6510436

[26] Cheng X Yang Y Schwebel DC Liu Z Li L Cheng P. Population ageing and mortality during 1990-2017: a global decomposition analysis. PLoS Med. (2020) 17:e1003138. doi: 10.1371/journal.pmed.1003138, PMID: 32511229 PMC7279585

[27] Schaffer AL Dobbins TA Pearson SA. Interrupted time series analysis using autoregressive integrated moving average (ARIMA) models: a guide for evaluating large-scale health interventions. BMC Med Res Methodol. (2021) 21:58. doi: 10.1186/s12874-021-01235-8, PMID: 33752604 PMC7986567

[28] Roth GA Mensah GA Johnson CO Addolorato G Ammirati E Baddour LM. Global burden of cardiovascular diseases and risk factors, 1990-2019: update from the GBD 2019 study. J Am Coll Cardiol. (2020) 76:2982–3021. doi: 10.1016/j.jacc.2020.11.010 33309175 PMC7755038

[29] Wang S Zhang C Zhang M Liang B Zhu H Lee J. Activation of AMP-activated protein kinase alpha 2 by nicotine instigates formation of abdominal aortic aneurysms in mice *in vivo*. Nat Med. (2012) 18:902–10. doi: 10.1038/nm.2711 22561688 PMC3559018

[30] Ghosh A Pechota A Coleman D Upchurch GR Jr Eliason JL. Cigarette smoke-induced MMP2 and MMP9 secretion from aortic vascular smooth cells is mediated via the Jak/stat pathway. Hum Pathol. (2015) 46:284–94. doi: 10.1016/j.humpath.2014.11.003, PMID: 25537973

[31] Jin J Arif B Garcia-Fernandez F Ennis TL Davis EC Thompson RW. Novel mechanism of aortic aneurysm development in mice associated with smoking and leukocytes. Arterioscler Thromb Vasc Biol. (2012) 32:2901–9. doi: 10.1161/ATVBAHA.112.300208, PMID: 23042818 PMC3506015

[32] Stackelberg O Bjorck M Larsson SC Orsini N Wolk A. Sex differences in the association between smoking and abdominal aortic aneurysm. Br J Surg. (2014) 101:1230–7. doi: 10.1002/bjs.9526, PMID: 24916023

[33] Raveendran M Wang J Senthil D Wang J Utama B Shen Y. Endogenous nitric oxide activation protects against cigarette smoking induced apoptosis in endothelial cells. FEBS Lett. (2005) 579:733–40. doi: 10.1016/j.febslet.2004.12.052, PMID: 15670837 PMC1350101

[34] Wang J Wilcken DE Wang XL. Cigarette smoke activates caspase-3 to induce apoptosis of human umbilical venous endothelial cells. Mol Genet Metab. (2001) 72:82–8. doi: 10.1006/mgme.2000.3115, PMID: 11161833

[35] Hsu CL Wu YL Tang GJ Lee TS Kou YR. *Ginkgo biloba* extract confers protection from cigarette smoke extract-induced apoptosis in human lung endothelial cells: role of heme oxygenase-1. Pulm Pharmacol Ther. (2009) 22:286–96. doi: 10.1016/j.pupt.2009.02.003, PMID: 19254777

[36] Cucina A Fuso A Coluccia P Cavallaro A. Nicotine inhibits apoptosis and stimulates proliferation in aortic smooth muscle cells through a functional nicotinic acetylcholine receptor. J Surg Res. (2008) 150:227–35. doi: 10.1016/j.jss.2007.10.019, PMID: 18295799

[37] Messner B Bernhard D. Smoking and cardiovascular disease: mechanisms of endothelial dysfunction and early atherogenesis. Arterioscler Thromb Vasc Biol. (2014) 34:509–15. doi: 10.1161/ATVBAHA.113.300156, PMID: 24554606

[38] Umebayashi R Uchida HA Wada J. Abdominal aortic aneurysm in aged population. Aging (Albany NY). (2018) 10:3650–1. doi: 10.18632/aging.101702, PMID: 30523221 PMC6326692

[39] G.B.D.T. Collaborators. Smoking prevalence and attributable disease burden in 195 countries and territories, 1990-2015: a systematic analysis from the global burden of disease study 2015. Lancet. (2017) 389:1885–906. doi: 10.1016/S0140-6736(17)30819-X, PMID: 28390697 PMC5439023

[40] Maimaitiming M Wang M Luo Y Wang J Jin Y Zheng ZJ. Global trends and regional differences in the burden of cancer attributable to secondhand smoke in 204 countries and territories, 1990-2019. Front Oncol. (2022) 12:972627. doi: 10.3389/fonc.2022.972627, PMID: 36303836 PMC9592919

[41] Wang Y Li Q Bi L Wang B Lv T Zhang P. Global trends in the burden of ischemic heart disease attributable to smoking from 1990 to 2021: a systematic analysis of the global burden of disease study 2021. Tob Induc Dis. (2025) 23:1–13. doi: 10.18332/tid/199931, PMID: 39882032 PMC11775718

[42] Song P He Y Adeloye D Zhu Y Ye X Yi Q. The global and regional prevalence of abdominal aortic aneurysms: a systematic review and modeling analysis. Ann Surg. (2023) 277:912–9. doi: 10.1097/SLA.0000000000005716 36177847 PMC10174099

[43] Lo RC Bensley RP Hamdan AD Wyers M Adams JE Schermerhorn ML. Gender differences in abdominal aortic aneurysm presentation, repair, and mortality in the vascular study Group of new England. J Vasc Surg. (2013) 57:1261–8, 1268.e1–5. doi: 10.1016/j.jvs.2012.11.039 23384493 PMC3633660

