# Supplementary material for: Global trends and inequities in smoking-attributable aortic aneurysm burden from 1990 to 2021 with future projections
Source: Front Public Health. 2025 Jul 29;13:1652544. doi: 10.3389/fpubh.2025.1652544 (PMC12339535; doi:10.3389/fpubh.2025.1652544)
Supplement: Supplementary file 1 [file Data_Sheet_1.docx]

Supplementary Material

# Supplementary Figures and Tables

## Supplementary Figures


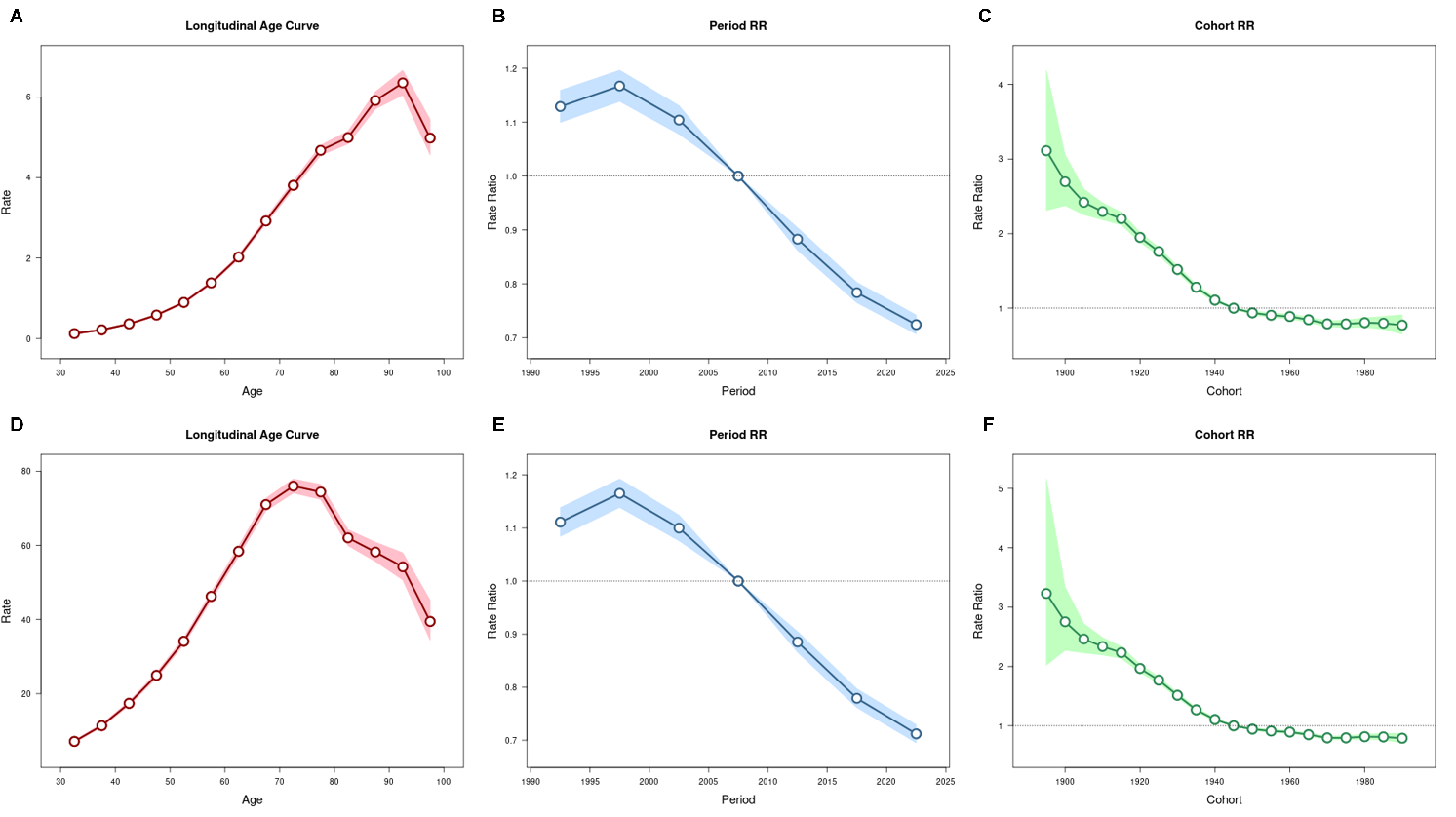


**Supplementary Figure 1.** The age-period-cohort analysis of AA burden attributable to smoking in death and DALY. The red curve is (A) mortality and (D) DALYs, disability-adjusted life years presents the relationship between longitudinal age and rate, the blue curve illustrates the relationship between period and rate ratio for (B) mortality and (E) DALYs, and the green curve depicts the relationship between cohort and rate ratio for (C) mortality and (F) DALYs.

## Supplementary Table1

**Table S1** Joinpoint analysis results for the trends in age-standardized mortality rates (ASMR) and age-standardized DALY rates (ASDR) of aortic aneurysms attributable to smoking by gender from 1990 to 2021.

| **Gender** | **Model** | **Segment** | **Segment. Start** | **Segment End** | **ASMR** | **lower** | **upper** | **P Value** | **APC** | **AAPC** |
| --- | --- | --- | --- | --- | --- | --- | --- | --- | --- | --- |
| Both | 3 | 0 | 1990 | 1994 | 0.98 | 0.51 | 1.45 | 0.000259 | 0.98 (0.51 - 1.45) | -1.80 (-1.89 - -1.72) |
| Both | 3 | 1 | 1994 | 2000 | -1.84 | -2.17 | -1.51 | <0.00001 | -1.84 (-2.17 - -1.51) |  |
| Both | 3 | 2 | 2000 | 2013 | -2.42 | -2.51 | -2.34 | <0.00001 | -2.42 (-2.51 - -2.34) |  |
| Both | 3 | 3 | 2013 | 2021 | -1.08 | -1.24 | -0.92 | <0.00001 | -1.08 (-1.24 - -0.92) |  |
| Female | 5 | 0 | 1990 | 1995 | 0.13 | -0.1 | 0.37 | 0.250454 | 0.13 (-0.10 - 0.37) | -2.11 (-2.24 - -1.99) |
| Female | 5 | 1 | 1995 | 2000 | -1.79 | -2.12 | -1.46 | <0.00001 | -1.79 (-2.12 - -1.46) |  |
| Female | 5 | 2 | 2000 | 2007 | -2.78 | -2.95 | -2.6 | <0.00001 | -2.78 (-2.95 - -2.60) |  |
| Female | 5 | 3 | 2007 | 2010 | -3.69 | -4.77 | -2.59 | <0.00001 | -3.69 (-4.77 - -2.59) |  |
| Female | 5 | 4 | 2010 | 2014 | -2.33 | -2.89 | -1.76 | <0.00001 | -2.33 (-2.89 - -1.76) |  |
| Female | 5 | 5 | 2014 | 2021 | -1.7 | -1.86 | -1.54 | <0.00001 | -1.70 (-1.86 - -1.54) |  |
| Male | 2 | 0 | 1990 | 1995 | 0.47 | 0.08 | 0.85 | 0.018607 | 0.47 (0.08 - 0.85) | -1.88 (-1.97 - -1.80) |
| Male | 2 | 1 | 1995 | 2013 | -2.38 | -2.44 | -2.32 | <0.00001 | -2.38 (-2.44 - -2.32) |  |
| Male | 2 | 2 | 2013 | 2021 | -1.06 | -1.23 | -0.88 | <0.00001 | -1.06 (-1.23 - -0.88) |  |
| **Gender** | **Model** | **Segment** | **Segment Start** | **Segment End** | **ASDR** | **lower** | **upper** | **P Value** | **APC** | **AAPC** |
| Both | 3 | 0 | 1990 | 1994 | 0.98 | 0.51 | 1.45 | 0.000259 | 0.98 (0.51 - 1.45) | -1.53 (-1.63 - -1.43) |
| Both | 3 | 1 | 1994 | 2000 | -1.84 | -2.17 | -1.51 | <0.00001 | -1.84 (-2.17 - -1.51) |  |
| Both | 3 | 2 | 2000 | 2013 | -2.42 | -2.51 | -2.34 | <0.00001 | -2.42 (-2.51 - -2.34) |  |
| Both | 3 | 3 | 2013 | 2021 | -1.08 | -1.24 | -0.92 | <0.00001 | -1.08 (-1.24 - -0.92) |  |
| Female | 5 | 0 | 1990 | 1995 | 0.13 | -0.1 | 0.37 | 0.250454 | 0.13 (-0.10 - 0.37) | -1.94 (-2.08 - -1.80) |
| Female | 5 | 1 | 1995 | 2000 | -1.79 | -2.12 | -1.46 | <0.00001 | -1.79 (-2.12 - -1.46) |  |
| Female | 5 | 2 | 2000 | 2007 | -2.78 | -2.95 | -2.6 | <0.00001 | -2.78 (-2.95 - -2.60) |  |
| Female | 5 | 3 | 2007 | 2010 | -3.69 | -4.77 | -2.59 | 0.000004 | -3.69 (-4.77 - -2.59) |  |
| Female | 5 | 4 | 2010 | 2014 | -2.33 | -2.89 | -1.76 | <0.00001 | -2.33 (-2.89 - -1.76) |  |
| Female | 5 | 5 | 2014 | 2021 | -1.7 | -1.86 | -1.54 | <0.00001 | -1.70 (-1.86 - -1.54) |  |
| Male | 2 | 0 | 1990 | 1995 | 0.47 | 0.08 | 0.85 | 0.018607 | 0.47 (0.08 - 0.85) | -1.58 (-1.66 - -1.51) |
| Male | 2 | 1 | 1995 | 2013 | -2.38 | -2.44 | -2.32 | <0.00001 | -2.38 (-2.44 - -2.32) |  |
| Male | 2 | 2 | 2013 | 2021 | -1.06 | -1.23 | -0.88 | <0.00001 | -1.06 (-1.23 - -0.88) |  |

## Supplementary Table2

**Table S2**. Joinpoint analysis results for the trends in age-standardized mortality rates (ASMR) and age-standardized DALY rates (ASDR) of aortic aneurysms attributable to smoking across different SDI regions from 1990 to 2021.

| **Location** | **Model** | **Segment** | **Segment Start** | **Segment End** | **ASMR** | **lower** | **upper** | **P Value** | **APC** | **AAPC** |
| --- | --- | --- | --- | --- | --- | --- | --- | --- | --- | --- |
| Global | 3 | 0 | 1990 | 1995 | 0.22 | -0.06 | 0.49 | 0.115777 | 0.22 (-0.06 - 0.49) | -1.53 (-1.63 - -1.43) |
| Global | 3 | 1 | 1995 | 2000 | -2.01 | -2.4 | -1.62 | <0.00001 | -2.01 (-2.40 - -1.62) |  |
| Global | 3 | 2 | 2000 | 2013 | -2.81 | -2.88 | -2.73 | <0.00001 | -2.81 (-2.88 - -2.73) |  |
| Global | 3 | 3 | 2013 | 2021 | -1.29 | -1.43 | -1.15 | <0.00001 | -1.29 (-1.43 - -1.15) |  |
| High-middle SDI | 1 | 0 | 1990 | 1994 | 4.57 | 3.15 | 6.02 | <0.00001 | 4.57 (3.15 - 6.02) | -0.42 (-0.63 - -0.21) |
| High-middle SDI | 1 | 1 | 1994 | 2021 | -1.22 | -1.3 | -1.15 | <0.00001 | -1.22 (-1.30 - -1.15) |  |
| High SDI | 3 | 0 | 1990 | 1996 | -0.89 | -1.14 | -0.65 | <0.00001 | -0.89 (-1.14 - -0.65) | -2.32 (-2.43 - -2.21) |
| High SDI | 3 | 1 | 1996 | 2002 | -2.78 | -3.12 | -2.45 | <0.00001 | -2.78 (-3.12 - -2.45) |  |
| High SDI | 3 | 2 | 2002 | 2013 | -4.09 | -4.21 | -3.97 | <0.00001 | -4.09 (-4.21 - -3.97) |  |
| High SDI | 3 | 3 | 2013 | 2021 | -1.7 | -1.88 | -1.52 | <0.00001 | -1.70 (-1.88 - -1.52) |  |
| Low-middle SDI | 3 | 0 | 1990 | 1995 | 1.71 | 1.31 | 2.11 | <0.00001 | 1.71 (1.31 - 2.11) | 0.97 (0.86 - 1.07) |
| Low-middle SDI | 3 | 1 | 1995 | 2012 | 0.78 | 0.71 | 0.84 | <0.00001 | 0.78 (0.71 - 0.84) |  |
| Low-middle SDI | 3 | 2 | 2012 | 2019 | 1.56 | 1.31 | 1.82 | <0.00001 | 1.56 (1.31 - 1.82) |  |
| Low-middle SDI | 3 | 3 | 2019 | 2021 | -0.23 | -1.57 | 1.13 | 0.730635 | -0.23 (-1.57 - 1.13) |  |
| Low SDI | 4 | 0 | 1990 | 1995 | 0.88 | 0.53 | 1.23 | 0.00005 | 0.88 (0.53 - 1.23) | 0.21 (0.05 - 0.37) |
| Low SDI | 4 | 1 | 1995 | 2005 | -1.3 | -1.44 | -1.16 | <0.00001 | -1.30 (-1.44 - -1.16) |  |
| Low SDI | 4 | 2 | 2005 | 2009 | -0.09 | -0.84 | 0.66 | 0.798768 | -0.09 (-0.84 - 0.66) |  |
| Low SDI | 4 | 3 | 2009 | 2015 | 2.51 | 2.18 | 2.84 | <0.00001 | 2.51 (2.18 - 2.84) |  |
| Low SDI | 4 | 4 | 2015 | 2021 | 0.31 | 0.07 | 0.55 | 0.013979 | 0.31 (0.07 - 0.55) |  |
| Middle SDI | 3 | 0 | 1990 | 1994 | 2.23 | 1.64 | 2.83 | <0.00001 | 2.23 (1.64 - 2.83) | -0.09 (-0.24 - 0.06) |
| Middle SDI | 3 | 1 | 1994 | 2000 | 0 | -0.38 | 0.38 | 0.998206 | -0.00 (-0.38 - 0.38) |  |
| Middle SDI | 3 | 2 | 2000 | 2014 | -0.94 | -1.02 | -0.86 | <0.00001 | -0.94 (-1.02 - -0.86) |  |
| Middle SDI | 3 | 3 | 2014 | 2021 | -0.1 | -0.3 | 0.11 | 0.346535 | -0.10 (-0.30 - 0.11) |  |
| **Location** | **Model** | **Segment** | **Segment Start** | **Segment End** | **ASDR** | **lower** | **upper** | **P Value** | **APC** | **AAPC** |
| Global | 3 | 0 | 1990 | 1994 | 0.98 | 0.51 | 1.45 | 0.000259 | 0.98 (0.51 - 1.45) | -1.80 (-1.89 - -1.72) |
| Global | 3 | 1 | 1994 | 2000 | -1.84 | -2.17 | -1.51 | <0.00001 | -1.84 (-2.17 - -1.51) |  |
| Global | 3 | 2 | 2000 | 2013 | -2.42 | -2.51 | -2.34 | <0.00001 | -2.42 (-2.51 - -2.34) |  |
| Global | 3 | 3 | 2013 | 2021 | -1.08 | -1.24 | -0.92 | <0.00001 | -1.08 (-1.24 - -0.92) |  |
| High-middle SDI | 1 | 0 | 1990 | 1994 | 4.41 | 2.75 | 6.1 | <0.00001 | 4.41 (2.75 - 6.10) | -0.49 (-0.67 - -0.31) |
| High-middle SDI | 1 | 1 | 1994 | 2021 | -1.11 | -1.2 | -1.02 | <0.00001 | -1.11 (-1.20 - -1.02) |  |
| High SDI | 3 | 0 | 1990 | 1995 | -0.63 | -1.02 | -0.24 | 0.002927 | -0.63 (-1.02 - -0.24) | -2.61 (-2.71 - -2.51) |
| High SDI | 3 | 1 | 1995 | 2002 | -2.48 | -2.78 | -2.18 | <0.00001 | -2.48 (-2.78 - -2.18) |  |
| High SDI | 3 | 2 | 2002 | 2013 | -3.7 | -3.85 | -3.56 | <0.00001 | -3.70 (-3.85 - -3.56) |  |
| High SDI | 3 | 3 | 2013 | 2021 | -1.32 | -1.52 | -1.11 | <0.00001 | -1.32 (-1.52 - -1.11) |  |
| Low-middle SDI | 3 | 0 | 1990 | 1996 | 1.54 | 1.28 | 1.8 | <0.00001 | 1.54 (1.28 - 1.80) | 1.04 (0.92 - 1.16) |
| Low-middle SDI | 3 | 1 | 1996 | 2014 | 0.73 | 0.68 | 0.78 | <0.00001 | 0.73 (0.68 - 0.78) |  |
| Low-middle SDI | 3 | 2 | 2014 | 2019 | 1.61 | 1.24 | 1.99 | <0.00001 | 1.61 (1.24 - 1.99) |  |
| Low-middle SDI | 3 | 3 | 2019 | 2021 | -0.18 | -1.29 | 0.94 | 0.741812 | -0.18 (-1.29 - 0.94) |  |
| Low SDI | 5 | 0 | 1990 | 1994 | 0.94 | 0.57 | 1.31 | <0.00001 | 0.94 (0.57 - 1.31) | 0.25 (0.11 - 0.38) |
| Low SDI | 5 | 1 | 1994 | 1997 | -0.3 | -1.44 | 0.84 | 0.578606 | -0.30 (-1.44 - 0.84) |  |
| Low SDI | 5 | 2 | 1997 | 2006 | -1.31 | -1.44 | -1.19 | <0.00001 | -1.31 (-1.44 - -1.19) |  |
| Low SDI | 5 | 3 | 2006 | 2009 | 0.29 | -0.81 | 1.4 | 0.58812 | 0.29 (-0.81 - 1.40) |  |
| Low SDI | 5 | 4 | 2009 | 2017 | 1.64 | 1.49 | 1.78 | <0.00001 | 1.64 (1.49 - 1.78) |  |
| Low SDI | 5 | 5 | 2017 | 2021 | 0.45 | 0.13 | 0.77 | 0.008975 | 0.45 (0.13 - 0.77) |  |
| Middle SDI | 5 | 0 | 1990 | 1994 | 2.37 | 1.84 | 2.9 | <0.00001 | 2.37 (1.84 - 2.90) | -0.16 (-0.28 - -0.05) |
| Middle SDI | 5 | 1 | 1994 | 2000 | -0.09 | -0.41 | 0.24 | 0.579337 | -0.09 (-0.41 - 0.24) |  |
| Middle SDI | 5 | 2 | 2000 | 2006 | -0.91 | -1.21 | -0.61 | 0.00001 | -0.91 (-1.21 - -0.61) |  |
| Middle SDI | 5 | 3 | 2006 | 2010 | -0.4 | -1.04 | 0.24 | 0.200599 | -0.40 (-1.04 - 0.24) |  |
| Middle SDI | 5 | 4 | 2010 | 2014 | -1.3 | -1.91 | -0.68 | 0.000459 | -1.30 (-1.91 - -0.68) |  |
| Middle SDI | 5 | 5 | 2014 | 2021 | 0.1 | -0.09 | 0.28 | 0.273776 | 0.10 (-0.09 - 0.28) |  |

## Supplementary Table3

| **Country** | **ASMR** | | **EAPC** | **Country** | **ASDR** | | **EAPC** |
| --- | --- | --- | --- | --- | --- | --- | --- |
|  | **1990** | **2021** |  |  | **1990** | **2021** |  |
| Montenegro | 3.21 (2.45-4.28) | 3.71 (2.55-4.99) | 0.68 (0.6 to 0.75) | Montenegro | 84.96 (66.76-108.35) | 91.45 (64.06-123.32) | 0.41 (0.32 to 0.5) |
| Armenia | 1.64 (1.34-2.06) | 3.59 (2.85-4.38) | 2.99 (2.53 to 3.45) | Armenia | 45.86 (37.83-57.26) | 87.06 (70.26-105.16) | 2.48 (2.04 to 2.93) |
| Denmark | 3.81 (3.3-4.31) | 1.89 (1.54-2.28) | -2.86 (-3.26 to -2.45) | Belarus | 34.34 (29.26-43.27) | 51.24 (40.12-64.42) | 0.80 (0.32 to 1.28) |
| Greece | 1.95 (1.71-2.23) | 1.87 (1.59-2.18) | -0.73 (-1.03 to -0.43) | Russian Federation | 29.16 (26.4-31.64) | 47.92 (40.77-54.45) | 1.33 (1 to 1.66) |
| Belarus | 1.18 (1-1.51) | 1.79 (1.4-2.25) | 0.94 (0.52 to 1.36) | Greece | 44.21 (39.55-49.48) | 44.75 (39.14-50.63) | -0.55 (-0.86 to -0.23) |
| Russian Federation | 0.98 (0.87-1.07) | 1.67 (1.41-1.92) | 1.53 (1.2 to 1.86) | Uruguay | 60.62 (52.95-68.14) | 41.09 (35.1-47.39) | -1.69 (-1.95 to -1.44) |
| Monaco | 2.07 (1.5-2.74) | 1.61 (1.11-2.31) | -0.83 (-0.99 to -0.68) | Serbia | 41.97 (33.03-51.56) | 40.59 (30.95-52.45) | -0.10 (-0.34 to 0.14) |
| Serbia | 1.69 (1.27-2.17) | 1.59 (1.21-2.08) | -0.1 (-0.34 to 0.13) | Nauru | 45.77 (32.89-64.14) | 38.48 (27.42-52.9) | -0.71 (-0.79 to -0.63) |
| Uruguay | 2.24 (1.9-2.56) | 1.56 (1.3-1.84) | -1.62 (-1.89 to -1.34) | Georgia | 6.78 (5.57-8.34) | 37.8 (30.34-46.79) | 8.34 (6.92 to 9.78) |
| Cyprus | 3.01 (2.1-4.24) | 1.51 (1.03-1.98) | -2.66 (-2.87 to -2.44) | Japan | 25.85 (22.9-28.8) | 37.43 (32.22-42.68) | 1.32 (1.23 to 1.41) |

**Table S3** The ASMR, ASDR and EAPC of the top 10 countries and territories with the highest smoking related-AA burdens in 1990 and 2021

## Supplementary Table4

Table S4 The ASMR, ASDR and EAPC of the bottom 10 countries and territories with lowest smoking-related AA burdens in 1990 and 2021.

| **Country** | **ASMR** | | **EAPC** | **Country** | **ASDR** | | **EAPC** |
| --- | --- | --- | --- | --- | --- | --- | --- |
|  | **1990** | **2021** |  |  | **1990** | **2021** |  |
| Saudi Arabia | 0.01 (0.01-0.02) | 0.04 (0.03-0.06) | 4.65 (4.12 to 5.18) | Saudi Arabia | 0.42 (0.27-0.61) | 1.35 (0.85-1.94) | 4.95 (4.38 to 5.52) |
| Afghanistan | 0.02 (0.01-0.04) | 0.08 (0.03-0.13) | 4.90 (4.55 to 5.24) | Sri Lanka | 2.36 (1.76-3.22) | 1.69 (1.03-2.61) | -0.93 (-1.03 to -0.83) |
| Sri Lanka | 0.11 (0.08-0.15) | 0.08 (0.05-0.12) | -0.87 (-1 to -0.74) | Niger | 2.11 (0.44-5.31) | 2 (0.36-4.95) | -0.38 (-0.52 to -0.23) |
| Tajikistan | 0.12 (0.08-0.16) | 0.08 (0.05-0.12) | -1.34 (-1.59 to -1.1) | Tajikistan | 3.13 (2.14-4.39) | 2.11 (1.37-3.01) | -1.39 (-1.59 to -1.2) |
| Ethiopia | 0.11 (0.03-0.23) | 0.09 (0.03-0.17) | -0.47 (-0.9 to -0.04) | Afghanistan | 0.64 (0.23-1.11) | 2.23 (1.02-3.79) | 4.88 (4.55 to 5.21) |
| Guatemala | 0.14 (0.11-0.18) | 0.09 (0.07-0.11) | -2.17 (-2.44 to -1.9) | Guatemala | 3.5 (2.85-4.24) | 2.27 (1.74-2.86) | -2.07 (-2.34 to -1.81) |
| Nicaragua | 0.1 (0.07-0.13) | 0.09 (0.07-0.12) | -0.10 (-0.26 to 0.05) | Nicaragua | 2.48 (1.87-3.18) | 2.29 (1.71-3.05) | -0.18 (-0.31 to -0.05) |
| Niger | 0.09 (0.02-0.22) | 0.09 (0.01-0.21) | -0.33 (-0.45 to -0.2) | Ethiopia | 2.95 (0.91-6.68) | 2.37 (0.87-4.44) | -0.75 (-1.2 to -0.31) |
| Morocco | 0.04 (0.02-0.06) | 0.1 (0.06-0.15) | 3.48 (3.28 to 3.68) | Sudan | 1 (0.42-2.52) | 2.78 (1.53-4.47) | 3.86 (3.61 to 4.11) |
| Sudan | 0.03 (0.01-0.08) | 0.1 (0.06-0.16) | 4.14 (3.89 to 4.4) | Morocco | 1.19 (0.63-1.83) | 2.86 (1.75-4.18) | 3.3 (3.1 to 3.5) |

## Supplementary Table5

**Table S5** Decomposition analysis of mortality for aortic aneurysm attributable to smoking for both gender in global and 5 Socio-Demographic Index (SDI) quintiles.

| **Location/Sex** | **Overll difference** | **Aging effect** | **population effect** | **Epidemiology change effect** | **Aging effect percent (%)** | **Population effect percent (%)** | **Epidemiology change effect (%)** |
| --- | --- | --- | --- | --- | --- | --- | --- |
| Low SDI | 4437.45 | 102.611 | 3748.367 | 586.473 | 2.31 | 84.47 | 13.22 |
| Low-middle SDI | 15718.18 | -411.988 | 10072.408 | 6057.758 | -2.62 | 64.08 | 38.54 |
| Middle SDI | 10844.06 | 1333.298 | 7399.48 | 2111.285 | 12.3 | 68.24 | 19.47 |
| High-middle SDI | 7458.86 | 1765.478 | 9964.636 | -4271.251 | 23.67 | 133.59 | -57.26 |
| High SDI | -6058.03 | 1077.521 | 12395.741 | -19531.295 | -17.79 | -204.62 | 322.4 |
| Global | 11052.61 | 7972.211 | 28232.499 | -25152.095 | 72.13 | 255.44 | -227.57 |
| Female | 1313.27 | 1486.498 | 5358.092 | -5531.321 | 113.19 | 408 | -421.19 |
| Male | 9739.35 | 7976.602 | 22853.943 | -21091.198 | 81.9 | 234.66 | -216.56 |

## Supplementary Table6

**Table S6**. Decomposition analysis of DALY for aortic aneurysm attributable to smoking for both gender in global and 5 Socio-Demographic Index (SDI) quintiles.

| **Location/Sex** | **Overall difference** | **Aging effect** | **population effect** | **Epidemiology change effect** | **Aging effect percent (%)** | **population effect percent (%)** | **Epidemiology change effect (%)** |
| --- | --- | --- | --- | --- | --- | --- | --- |
| Low SDI | 21917.11 | -1001.489 | 21299.119 | 1619.485 | -4.57 | 97.18 | 7.39 |
| Low-middle SDI | 107944.77 | 5510.673 | 73520.934 | 28913.163 | 5.11 | 68.11 | 26.79 |
| Middle SDI | 168641.08 | 25142.498 | 149917.146 | -6418.563 | 14.91 | 88.9 | -3.81 |
| High-middle SDI | 137880.98 | 14404.89 | 206512.706 | -83036.619 | 10.45 | 149.78 | -60.22 |
| High SDI | -233561.27 | 61498.054 | 308223.315 | -603282.643 | -26.33 | -131.97 | 258.3 |
| Global | 288185.61 | 127597.823 | 674894.643 | -514306.855 | 44.28 | 234.19 | -178.46 |
| Female | 28078.12 | 21452.654 | 117375.845 | -110750.377 | 76.4 | 418.03 | -394.44 |
| Male | 260107.49 | 127985.919 | 555509.071 | -423387.502 | 49.21 | 213.57 | -162.77 |
